# Supplementary material for: Quasi-perfusion studies for intensified lentiviral vector production using a continuous stable producer cell line
Source: Mol Ther Methods Clin Dev. 2024 May 7;32(2):101264. doi: 10.1016/j.omtm.2024.101264 (PMC11141457; doi:10.1016/j.omtm.2024.101264)
Supplement: Document S1. Figures S1 and S2 [file mmc1.pdf]

OMTM, Volume *32*

Supplemental information

Quasi-perfusion studies for intensified lentiviral vector production using a continuous stable producer cell line

Dale J. Stibbs, Pedro Silva Couto, Yasuhiro Takeuchi, Qasim A. Rafiq, Nigel B. Jackson, and Andrea C. M. E. Rayat

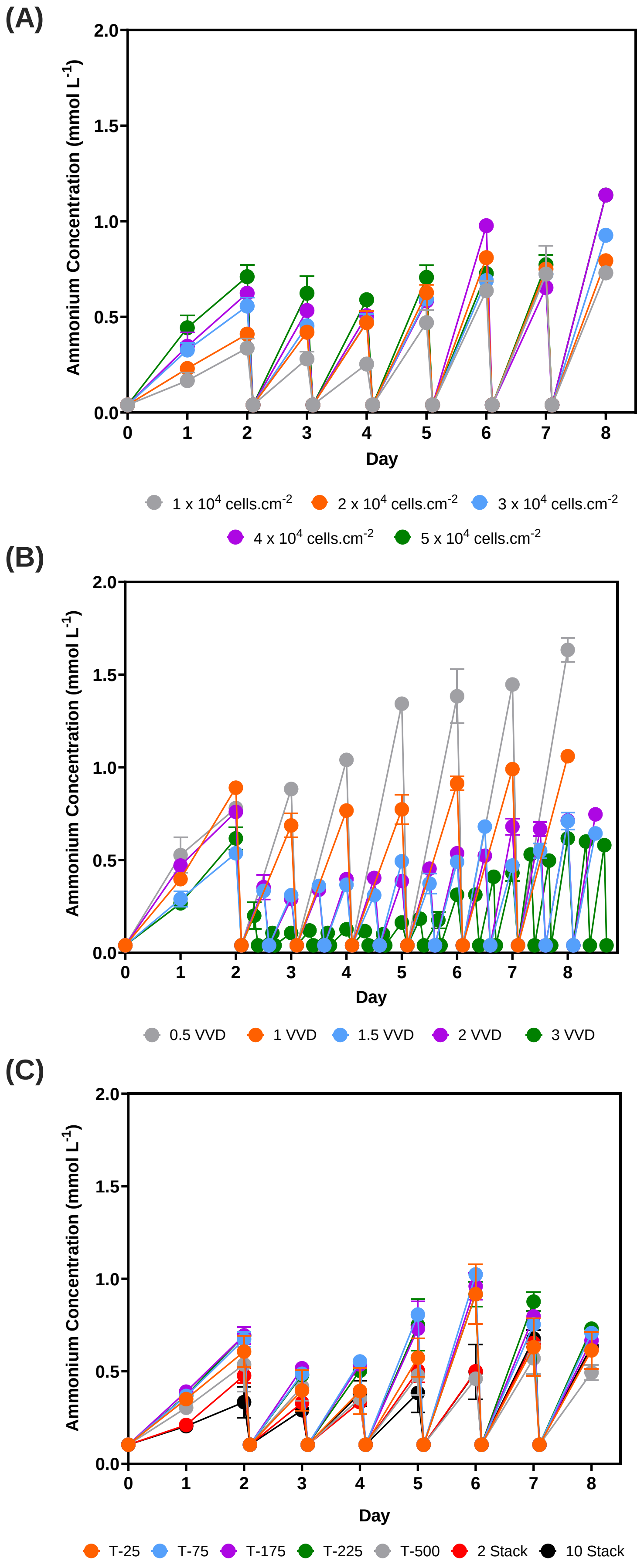
(A)
Ammonium Concentration (mmol L-1)
Day
1 x 10^4 cells.cm^-2
2 x 10^4 cells.cm^-2
3 x 10^4 cells.cm^-2
4 x 10^4 cells.cm^-2
5 x 10^4 cells.cm^-2
(B)
Ammonium Concentration (mmol L-1)
Day
0.5 VVD
1 VVD
1.5 VVD
2 VVD
3 VVD
(C)
Ammonium Concentration (mmol L-1)
Day
T-25
T-75
T-175
T-225
T-500
2 Stack
10 Stack

Figure S1 – Comparison of daily off-line measurements of ammonia during **(A)** the seeding density experiment where WinPac-RDpro-GFP cells were seeded at 1, 2, 3, 4 and 5 x 10^4 cells cm^{-2} and expanded in T-25 flasks for 8 days with a quasi-perfusion rate of 1 VVD commencing 48 hours post-seeding, **(B)** the quasi-perfusion experiment where WinPac-RDpro-GFP cells were seeded at 3 x 10^4 cells cm^{-2} were expanded in T-25 flasks for eight days with quasi-perfusion rates of 0.5, 1, 1.5, 2 and 3 VVD commencing 48 hours post-seeding and **(C)** the scaling experiment where WinPac-RDpro-GFP cells were seeded at 3 x 10^4 cells cm^{-2} were expanded in T-25, T-75, T-175, T-225, T-500 flasks and 2- and 10-layer multilayer flasks for eight days with quasi-perfusion rates of 0.5, 1, 1.5, 2 and 3 VVD commencing 48 hours post-seeding. Points represent mean value ± one standard deviation (N = 3).

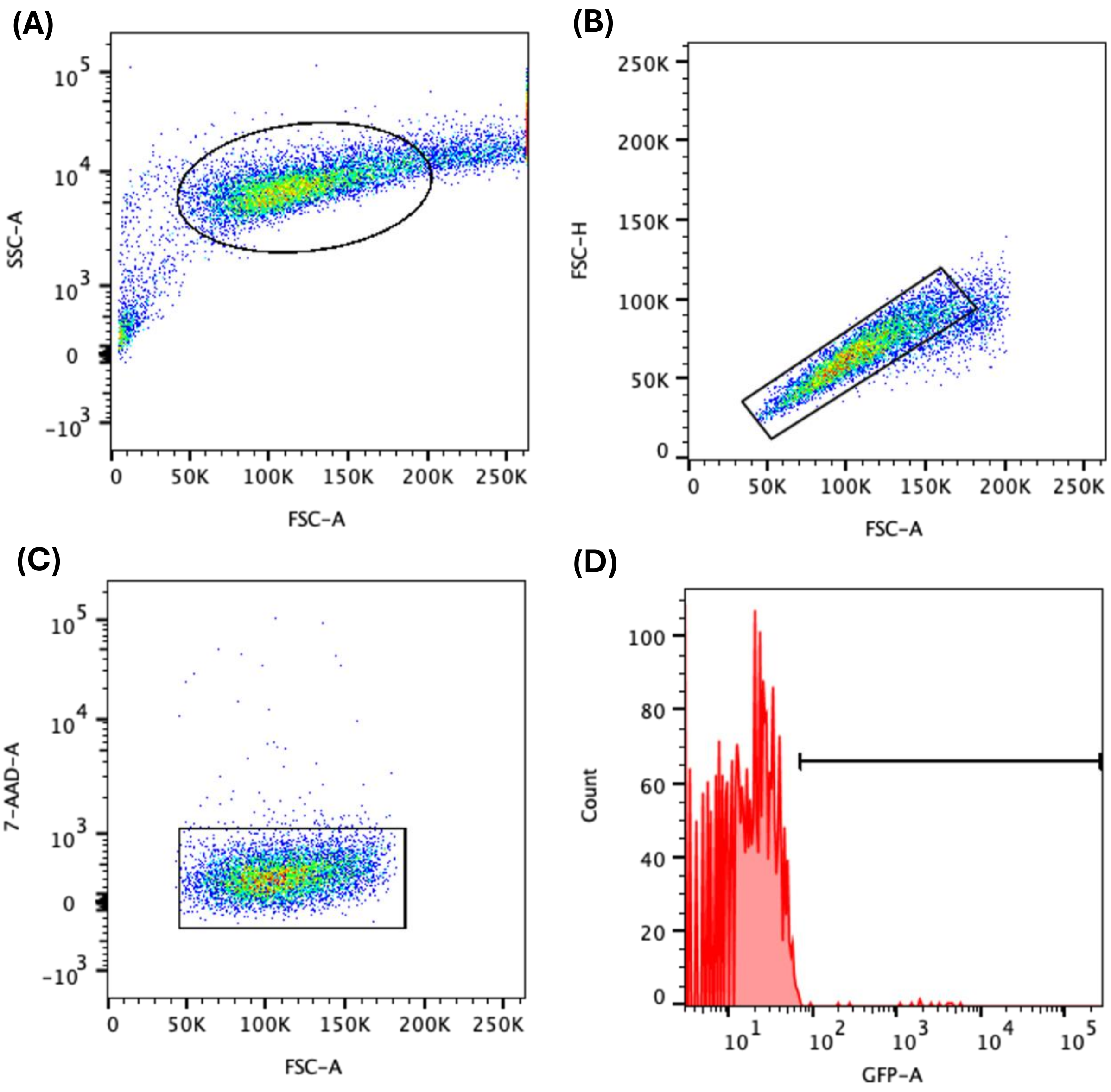

Figure S2 – Gating strategy for determining the percentage of GFP-expressing cells when tittering LVs using HEK 293T cells. **(A)** Gating of cells from debris; **(B)** Gating of singlets and doublets; **(C)** Gating of live cells through staining with 7-AAD; and **(D)** Gating of GFP-expressing cells.
