# Supplementary material for: Quasi-perfusion studies for intensified lentiviral vector production using a continuous stable producer cell line
Source: Mol Ther Methods Clin Dev. 2024 May 7;32(2):101264. doi: 10.1016/j.omtm.2024.101264 (PMC11141457; doi:10.1016/j.omtm.2024.101264)
Supplement: Document S2. Article plus supplemental information [file mmc2.pdf]

Molecular Therapy
Methods & Clinical Development
Original Article

Quasi-perfusion studies for intensified lentiviral vector production using a continuous stable producer cell line

Dale J. Stibbs,[1] Pedro Silva Couto,[1] Yasuhiro Takeuchi,[2,3] Qasim A. Rafiq,[1] Nigel B. Jackson,[4] and Andrea C. M. E. Rayat[1]

[1]Department of Biochemical Engineering, University College London, Bernard Katz Building, Gower Street, London WC1E 6BT, UK; [2]Division of Infection and Immunity, University College London, Cruciform Building, Gower Street, London WC1E 6BT, UK; [3]Biotherapeutics and Advanced Therapies, Scientific Research and Innovation, Medicines and Healthcare products Regulatory Agency, South Mimms EN6 3QC, Potters Bar, UK; [4]Cytiva, 5 Harbourgate Business Park, Southampton Road, Portsmouth PO6 4BQ, UK

Quasi-perfusion culture was employed to intensify lentiviral vector (LV) manufacturing using a continuous stable producer cell line in an 8-day process. Initial studies aimed to identify a scalable seeding density, with 3, 4, and 5 $\times$ 10^4 cells cm^{-2} providing similar specific productivities of infectious LV. Seeding at 3 $\times$ 10^4 cells cm^{-2} was selected, and the quasi-perfusion was modulated to minimize inhibitory metabolite accumulation and vector exposure at 37°C. Similar specific productivities of infectious LV and physical LV were achieved at 1, 2, and 3 vessel volumes per day (VVD), with 1 VVD selected to minimize downstream processing volumes. The optimized process was scaled 50-fold to 1,264 cm^2 flasks, achieving similar LV titers. However, scaling up beyond this to a 6,320 cm^2 multilayer flask reduced titers, possibly from suboptimal gas exchange. Across three independent processes in 25 cm^2 to 6,320 cm^2 flasks, reproducibility was high with a coefficient of variation of 7.7% ± 2.9% and 11.9% ± 3.0% for infectious and physical LV titers, respectively. The optimized flask process was successfully transferred to the iCELLis Nano (Cytiva) fixed-bed bioreactor, with quasi-perfusion at 1 VVD yielding 1.62 $\times$ 10^8 TU.

INTRODUCTION

Lentiviral vectors (LVs) play a pivotal role in gene-modified cell therapy manufacturing by facilitating stable gene expression in various cells, including T cells, hematopoietic stem cells, and mesenchymal stem cells.[1,2] LV-based therapies have demonstrated clinical efficacy in treating Wiskott-Aldrich syndrome, X-linked severe combined immunodeficiency, and beta-thalassemia.[3–5] However, their prohibitive costs may preclude the widespread adoption of these therapies. LV manufacturing contributes substantially to the overall costs, mainly due to the modest process yields obtained and the expensive plasmid DNA and transfection reagent.[6]

Transient transfection is the dominant LV manufacturing approach. Once optimized, it provides high unconcentrated titers and offers process flexibility by allowing production to pivot to produce vectors with different transgenes and envelope proteins.[7] However, there is a desire to use stable producer cell lines, which could facilitate more reproducible and scalable manufacturing while decreasing processing costs.[8–10] Developing these cell lines has proved challenging due to the cytotoxicity of the commonly used envelope protein vesicular stomatitis virus G protein (VSV-G) and HIV 1 protease.[11–18] An alternative approach is to use continuous packaging cell lines that maintain the expression of all vector components without any limitations on cytotoxicity, which provides an extended harvest window. This work uses the WinPac-RDpro-GFP cells that continuously express third-generation LVs pseudotyped with the RD114-Pro envelope protein, which efficiently transduces hematopoietic stem cells and T cells.[19]

Although recent interest has been in adopting suspension systems for LV production, many existing cell lines are adherent. Therefore, there remains a need for increased knowledge on manufacturing and scale-up of LV production using these cell lines. Small-scale LV production is conducted in culture flasks or well plates.[7] Process scale-up involves using vessels with larger surface areas, adding supplementary vessels, or using a fixed-bed bioreactor (FBR).[10,20–25] Multilayer flasks provide an efficient solution with vertically stacked, interconnected layers to provide large surface areas.[26,27] FBRs offer an alternative to surface-providing culture systems and microcarrier suspension culture in a controlled, low-shear environment for cell expansion.[28] These closed, single-use vessels feature a three-dimensional-like matrix with porous microfiber carriers or disks for cell adherence and proliferation. In contrast with static culture systems, FBRs significantly decrease the process footprint for the equivalent production capacity. These also provide a straightforward approach to scale-up from culture flasks,

Received 19 January 2024; accepted 3 May 2024;
https://doi.org/10.1016/j.omtm.2024.101264.

Correspondence: Andrea C. M. E. Rayat, Department of Biochemical Engineering, Bernard Katz Building, University College London, London WC1E 6BT, UK.
E-mail: andrea.rayat@ucl.ac.uk

© 2024 The Authors.
This is an open access article under the CC BY license (http://creativecommons.org/licenses/by/4.0/).

with iCELLis (Cytiva) and scale-X (Univercells Technologies by Donaldson) platforms providing surface areas of 0.53–500 m^2 and 2.4–600 m^2, respectively.[20–23]

An advantage of adherent culture is that it facilitates medium exchange and harvest of the LVs from the culture medium. Perfusion and quasi-perfusion approaches can intensify LV manufacturing by achieving higher cell densities by adding nutrients and by-product removal. Additionally, losses of infectious LV due to the short half-life ($t_{1/2}$) at 37°C can be minimized. Optimization of the (quasi-)perfusion rate is necessary to maximize the infectious LV titer while minimizing the volumes for downstream processing (DSP). There are many benefits to a fully continuous bioprocess that employs perfusion culture. However, perfusion processing requires specific bioreactors and control configurations. In contrast, quasi-perfusion approaches can be applied to multilayer flasks and bioreactors, enabling process intensification using these different systems. An additional challenge when scaling up adherent culture processes is generating sufficient cell numbers for seeding. It is, therefore, desirable to minimize the seeding density to shorten the seed train and overall process duration.

This study established an intensified LV manufacturing process by implementing quasi-perfusion processing with a continuous stable producer cell line to harvest vectors over an extended period. A scalable process was first developed at small-scale in T-25 flasks by optimizing the seeding density and quasi-perfusion rate. Next, the impact of process scale-up from T-25 flasks to multilayer flasks with a surface area of 6,320 cm^2 on batch-to-batch variability, vector titers and supernatant quality as measured by the ratio of physical-to-infectious LVs was evaluated. The optimized process was transferred to a FBR and operated in quasi-perfusion mode.

RESULTS

Impact of seeding density on LV production

A process for manufacturing LVs using the stable producer cell line, WinPac-RDpro-GFP, was first established at small scale using T-25 flasks. The initial study assessed the impact on cell growth kinetics, LV titers, and metabolite consumption and production of varying the seeding density between 1×10^4 and 5×10^4 cells cm^{-2}.

As shown in Figure 1A, significant cell detachment was observed on the final day at the higher seeding densities. The cell viability remained above 90% throughout the process (Figure 1A). Population doublings decreased with increasing seeding densities until 4×10^4 cells cm^{-2}, after which a plateau was observed (Table 1). Specific growth rates were similar across seeding densities (0.020 ± 0.002) h^{-1}. The cell diameter increased during the first 2 days of culture (Figure 1B). A decrease was observed at all seeding densities during the exponential and stationary phases.

Analysis of infectious LV titers showed seeding densities of 3, 4, and 5×10^4 cells cm^{-2} produced similar total specific productivities of infectious LV (Figure 2A). The total specific productivities of physical LVs were consistent across all seeding densities, ranging from 236 to 274 vp $cell^{-1}$ (Figure 2C). The process with a seeding density of 1×10^4 cells cm^{-2} has a significantly higher ratio of physical-to-infectious LVs calculated based on the specific productivities at 778 ± 32 compared with the higher densities (Figure 2E). The 4×10^4 and 5×10^4 cells cm^{-2} seeding densities produced the highest quality supernatant at 368 ± 11 and 246 ± 1, respectively.

Figure 1C shows that seeding densities of 2, 3, 4, and 5×10^4 cells cm^{-2} led to similar glucose concentration profiles that closely tracked the viable cell densities. Higher glucose concentrations were maintained in the 1×10^4 cells cm^{-2}. Lactate concentrations generally increased with higher cell densities, with concentrations greater than 25 mmol L^{-1} observed on day 8 (Figure 1D). Ammonium concentrations remained below 1.2 mmol L^{-1} (Figure S1A). Figure 1E shows the daily lactate yield from glucose was approximately 2 mol mol^{-1}, except on day 3. For seeding densities of 2, 3, 4, and 5×10^4 cells cm^{-2}, the lowest pH values of 6.37 ± 0.28 to 6.24 ± 0.07 were recorded on days 7 and 8, respectively (Figure 1F). Conversely, the process seeded with 1×10^4 cells cm^{-2} maintained a pH above 7.00.

Half-life of RDpro-pseudotyped LV

The $t_{1/2}$ of RDpro-pseudotyped LVs in culture medium was determined at 4°C, 21°C, and 37°C (Figure 3). The $t_{1/2}$ values were 16.6 ± 1.2 hours at 37°C, 18.8 ± 3.3 hours at 21°C, and 24.2 ± 0.4 hours. All temperatures exhibited a one-phase exponential decay.

Impact of quasi-perfusion rate on LV production

Modulating the quasi-perfusion rate can curb inhibitory metabolite accumulation, prevent nutrient depletion, and minimize losses through low vector stability at 37°C. The impact of varying the quasi-perfusion rate between 0.5 and 3 vessel volumes per day (VVD) on cell growth kinetics, LV titers, and metabolite consumption and production was evaluated.

As shown in Table 1, similar population doublings were observed at 0.5, 1, 2, and 3 VVD at 3.9 ± 0.2. During the 1.5 VVD process, detachment of cells was observed from day 3 despite confluency not being attained (Figure 4A). This was reflected in the population doublings, which were 2.5 ± 0.1. The doubling times were the longest in the 0.5 VVD process at 43.3 ± 1.7 h, with the remaining quasi-perfusion rates averaging 36.1 ± 2.4 h. The cell viability was maintained above 90% for the duration of the process (Figure 4A). The cell diameter increased for the first 2 days before decreasing for the remainder of the process (Figure 4B).

As with the previous study, glucose depletion did not occur (Figure 4C). The 0.5 VVD process resulted in a lactate concentration profile comparable with the 1 VVD process (Figure 4D). The 1.5 VVD process resulted in the lowest lactate concentrations on days 6, 7, and 8. Quasi-perfusion rates of 2 and 3 VVD reduced the lactate accumulation relative to the 1 VVD process. The processes at 0.5, 1, and 2 VVD resulted in a daily lactate yield from glucose close to the theoretical maximum value of 2 mol mol^{-1} (Figure 4E). Yields below

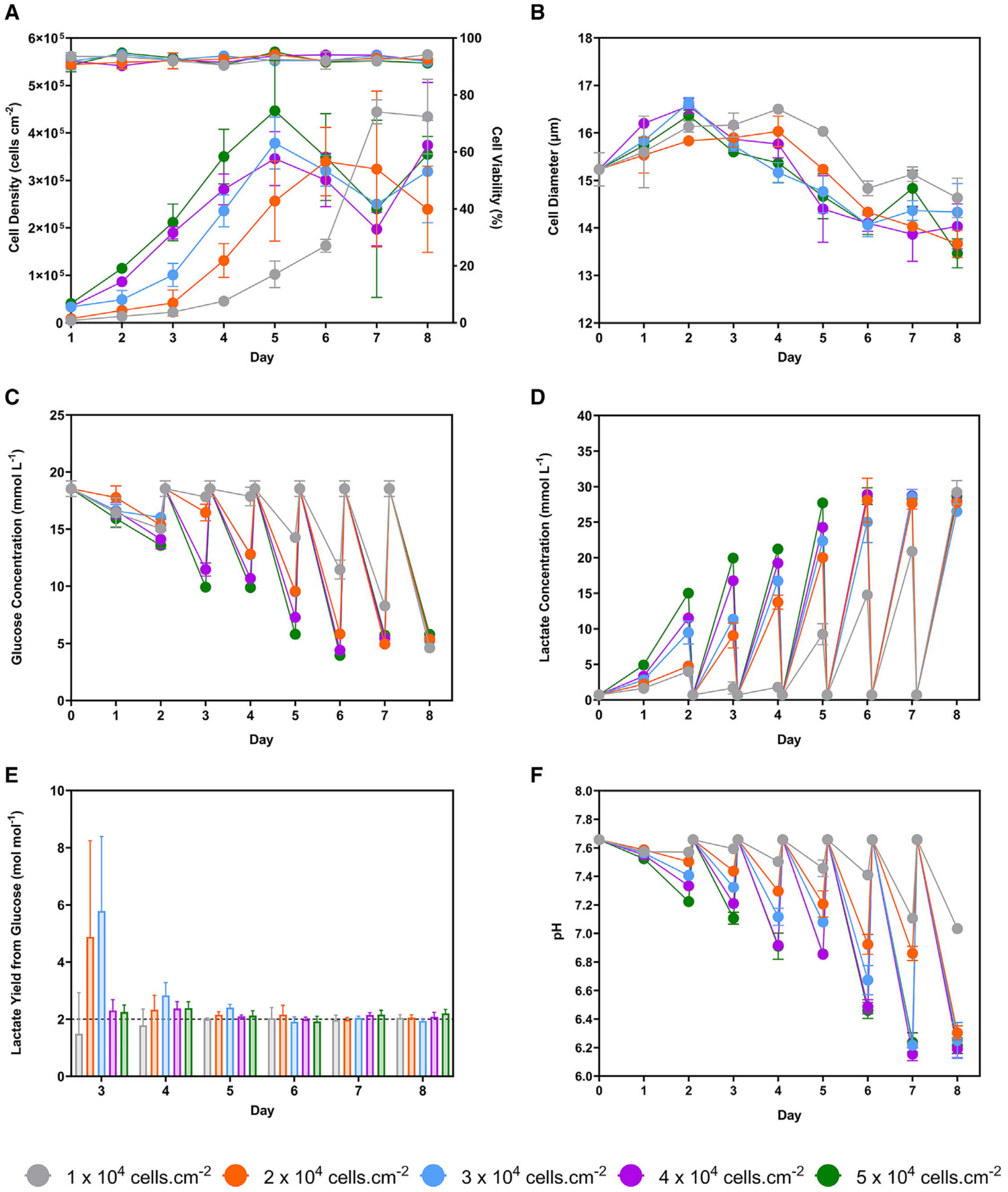

Figure 1. Cell culture profile during the seeding density study for LV production

(A) Viable WinPac-RDpro-GFP cell number and (B) cell diameter at each seeding density ($n = 3$). Daily offline measurement of (C) glucose and (D) lactate at each seeding density ($n = 3$). (F) The pH at each seeding density ($n = 1$). (E) The lactate yield from glucose at each seeding density from days three to eight ($n = 3$). Data points and bars represent mean value ± one standard deviation (N = 3). The 2 mol mol^{-1} reference line indicates the maximum theoretical yield of lactate from glucose during anaerobic glycolysis. WinPac-RDpro-GFP cells were seeded at 1, 2, 3, 4, and 5 × 10^4 cells cm^{-2} and expanded in T-25 flasks for 8 days with a quasi-perfusion rate of 1 VVD commencing 48 h after seeding.

the maximum theoretical value were obtained at 1.5 and 3 VVD quasi-perfusion rates. Ammonium concentrations did not exceed 1.7 mmol L^{-1} and were generally below 1.0 mmol L^{-1} (Figure S1B).

Offline pH measurements indicated that the medium became more acidic as the process progressed (Figure 4F), with the 0.5 and 1 VVD process recording values of 6.44 ± 0.02 and 6.28 ± 0.06 on

Table 1. Impact of seeding density and quasi-perfusion rate on the specific growth rate, doubling time and population doublings

Seeding density (cells cm^{-2})	Specific growth rate (h^{-1})	Doubling time (h)	Population doublings
1×10^4	0.023 ± 0.001	30.7 ± 0.4	5.5 ± 0.1
2×10^4	0.020 ± 0.001	34.6 ± 1.1	4.4 ± 0.2
3×10^4	0.021 ± 0.001	33.0 ± 1.5	3.7 ± 0.2
4×10^4	0.018 ± 0.001	38.8 ± 2.3	3.1 ± 0.2
5×10^4	0.020 ± 0.001	34.9 ± 1.7	3.2 ± 0.2
Quasi-perfusion rate (VVD)	Specific growth rate (h^{-1})	Doubling time (h)	Population doublings
0.5	0.016 ± 0.001	43.3 ± 1.7	3.9 ± 0.2
1	0.020 ± 0.001	34.5 ± 1.9	3.9 ± 0.2
1.5	0.018 ± 0.001	37.9 ± 1.2	2.5 ± 0.1
2	0.020 ± 0.001	34.7 ± 2.0	3.9 ± 0.1
3	0.019 ± 0.001	37.4 ± 1.9	3.9 ± 0.2

For the seeding density experiment, WinPac-RDpro-GFP cells were seeded at 1, 2, 3, 4 and 5×10^4 cells cm^{-2} in T-25 flasks and expanded for 8 days with quasi-perfusion at 1 VVD commencing 2 days after seeding. During the quasi-perfusion experiment, WinPac-RDpro-GFP cells were seeded at 3×10^4 cells cm^{-2} in T-25 flasks and expanded for 8 days with quasi-perfusion at 0.5, 1, 1.5, 2, and 3 VVD starting 2 days after seeding. Values represent mean ± one standard deviation ($N = 3$).

day 8. In contrast, the 2 and 3 VVD processes mostly maintained a pH above 7.0.

Higher quasi-perfusion rates did not increase the total specific productivities of infectious LVs. At 2 and 3 VVD, the specific productivities were similar to the 1 VVD process (Figure 2B). All rates yielded comparable total specific productivities of physical LVs (Figure 2D). The 0.5 VVD process produced the lowest quality supernatant with a physical-to-infectious LV ratio calculated based on the specific productivities of 851 ± 22. Conversely, the ratio at 1 VVD was 357 ± 8, indicating the highest quality supernatant (Figure 2F). As a quasi-perfusion rate of 1 VVD maximized the infectious titers and minimized the process volumes to facilitate DSP, this was carried forward in subsequent studies.

Scale-up of the optimized process

The process with a seeding density of 3×10^4 cells cm^{-2} and a quasi-perfusion rate of 1 VVD was scaled from T-25 flasks to flasks with a surface area of 75, 175, and 225 cm^2 and multilayer flasks, with surface areas of 500, 1,264, and 6,320 cm^2.

As seen in Figure 5A, infectious LV titers were comparable when scaling up from 25 cm^2 to 1,264 cm^2. The infectious titers achieved in the 6,320 cm^2 flasks were $(1.36 \pm 0.13) \times 10^5$ TU cm^{-2}, about 1.6-fold lower than the $(2.24 \pm 0.25) \times 10^5$ TU cm^{-2} achieved in the flasks with surface areas ranging from 25 to 1,264 cm^2. Similarly, the physical LV titers in 6,320 cm^2 flasks were lower at $(2.17 \pm 0.24) \times 10^7$ vp cm^{-2}, while the flasks with surface areas of 25 cm^2 to 1,264 cm^2 averaged $(5.22 \pm 0.78) \times 10^7$ vp cm^{-2} (Figure 5B). Figure 5C shows similar ratios of physical-to-infectious LVs were observed across all the flasks at 241 ± 39. The batch-to-batch variability remained low when scaling up, with the infectious and physical LV titer coefficient of variation (CV) being 7.7% ± 2.6% and 11.9% ± 3.0%, respectively, across three independent processes for all the flasks.

The lowest glucose concentration in the 6,320 cm^2 multilayer flask was 9.51 ± 0.42 mmol L^{-1} on day 8, while the flasks with smaller surface areas had 5.52 ± 0.62 mmol L^{-1} at the same time (Figure 6A). Lactate concentrations peaked at 27.27 ± 2.0 mmol L^{-1} in the T-25, T-75, T-175, T-225, and T-500 flasks at day 6 (Figure 6B). In contrast, the 1,264 cm^2 and 6,320 cm^2 flasks increased until day 7, after which the lactate concentration plateaued. The average lactate yield from glucose was 1.62 ± 0.24 mol mol^{-1}, which is lower than the maximum theoretical value of approximately 2 mol mol^{-1} (Figure 6C). The ammonium concentrations in the flasks ranging from 25 to 500 cm^2 peaked on day 6 at 0.86 ± 0.21 mmol L^{-1} (Figure S1C). The 1,264 cm^2 and 6,320 cm^2 multilayer flasks showed a more gradual increase in ammonium concentrations, with the maximum observed on days 7 and 8 at 0.67 ± 0.03 mmol L^{-1} and 0.63 ± 0.02 mmol L^{-1}. The culture pH in the flasks with surface areas from 25 to 500 cm^2 closely tracked each other. The pH decreased until a plateau was reached on days 7 and 8, where the concentrations were 6.13 ± 0.13 and 6.33 ± 0.09, respectively (Figure 6D). In contrast, a gradual decrease in pH was observed in the 1,264 and 6,320 cm^2 multilayer flasks. In these flasks, the lowest pH was observed on day 8 at 6.62 ± 0.03.

Scaling up the process did not impact the double-stranded DNA (dsDNA) produced per cm^2, with the average being 188 ± 38 ng cm^{-2} (Figure 6E). Across the flasks, harvested dsDNA concentrations were similar on days 3, 4, 5, and 6 at 26 ± 11 ng cm^{-2}. This increased significantly on days 7 and 8 to 39 ± 13 ng cm^{-2} and 46 ± 12 ng cm^{-2}, respectively (Figure 6F).

Process transfer to FBRs

After scaling up to multilayer flasks, the process was transferred to the iCELLis Nano FBR. A seeding density of 3×10^4 cells cm^{-2} was maintained when transferring systems. The medium exchange was performed at 1 VVD in quasi-perfusion mode.

The specific growth rate was 0.016 and 0.015 h^{-1} in the quasi-perfusion and continuous processes, respectively (Table 2). In addition, the number of population doublings increased from 3.3 during the quasi-perfusion process to 3.7 in the continuous process in a recent work.[10]

DISCUSSION

LVs are critical in manufacturing gene-modified cell therapies, as they can efficiently transduce dividing and non-dividing cells to provide stable gene expression. They are typically manufactured by co-transfecting mammalian cells with plasmid DNA coding for the vector genome.[7] Chemical transfection achieves high LV titers once optimized and provides flexibility to pivot production to

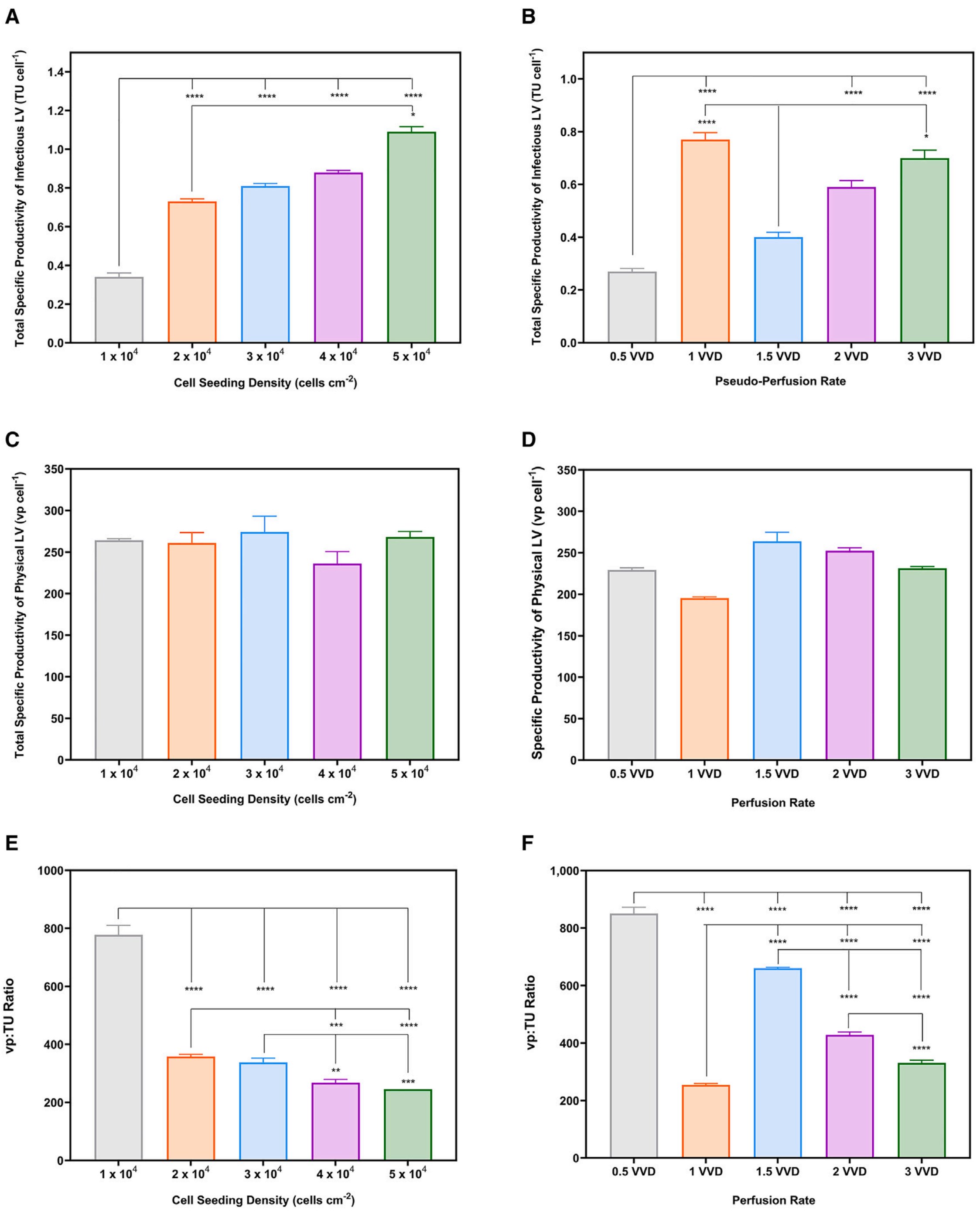

Figure 2. LV titers and product quality for seeding density and quasi-perfusion studies

Total specific productivities of infectious LV during (A) the seeding density and (B) the quasi-perfusion study (n = 6). Total specific productivities of physical LV during (C) the seeding density and (D) the quasi-perfusion study (n = 3). Infectious titers were determined using flow cytometric detection of GFP-expressing cells. The ratio of physical-to-infectious LVs was calculated based on the specific productivities for (E) the seeding density and (F) the quasi-perfusion process. During the seeding density study, WinPac-RDpro-GFP cells were seeded at 1, 2, 3, 4, and 5 $\times$ 10^4 cells cm^{-2} and expanded in T-25 flasks for 8 days with a quasi-perfusion rate of 1 VVD commencing 48 h after seeding. In the quasi-perfusion study, WinPac-RDpro-GFP cells were seeded at 3 $\times$ 10^4 cells cm^{-2} and were expanded in T-25 flasks for 8 days with quasi-perfusion rates of 0.5, 1, 1.5, 2, and 3 VVD commencing 48 h after seeding. Error bars represent mean value ± one standard deviation (N = 3).

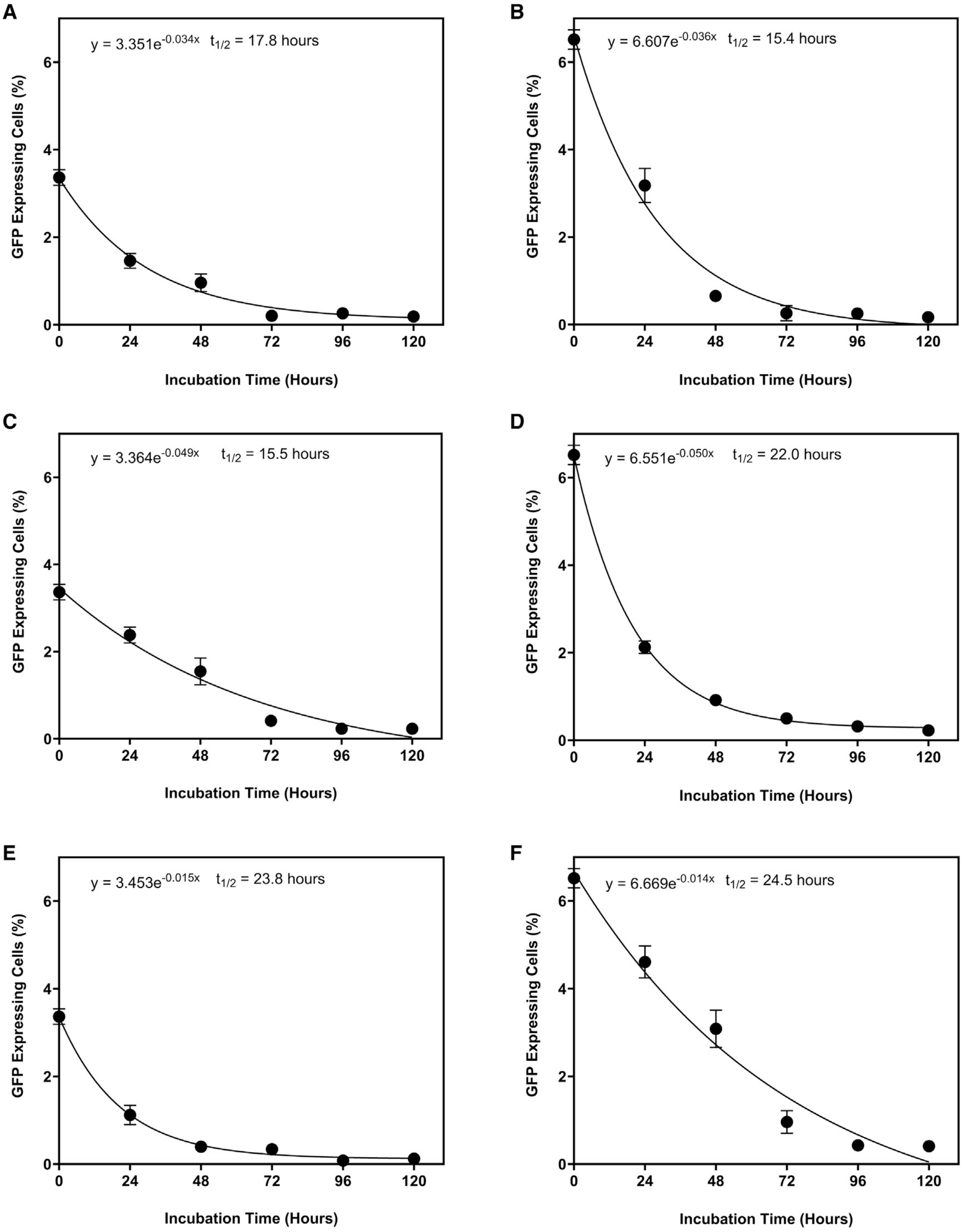

Figure 3. Half-life of RDpro pseudotyped LVs at different temperatures

Half-life of RDpro-pseudotyped LVs in DMEM with 10% (v/v) FBS (pH 7.20). LVs were incubated at temperatures of 37°C (A and B), 21°C (C and D), and 4°C (E and F) ($N = 2$). The two results at each temperature represent two independent $t_{1/2}$ experiments with different batches of LVs. LVs were produced using the WinPac-RDpro-GFP stable cell line. Points represent mean value ± one standard deviation ($n = 7$). A flow cytometry dot plot is provided in Figure S2 to show the gating approach for determining the infectious LV titer.

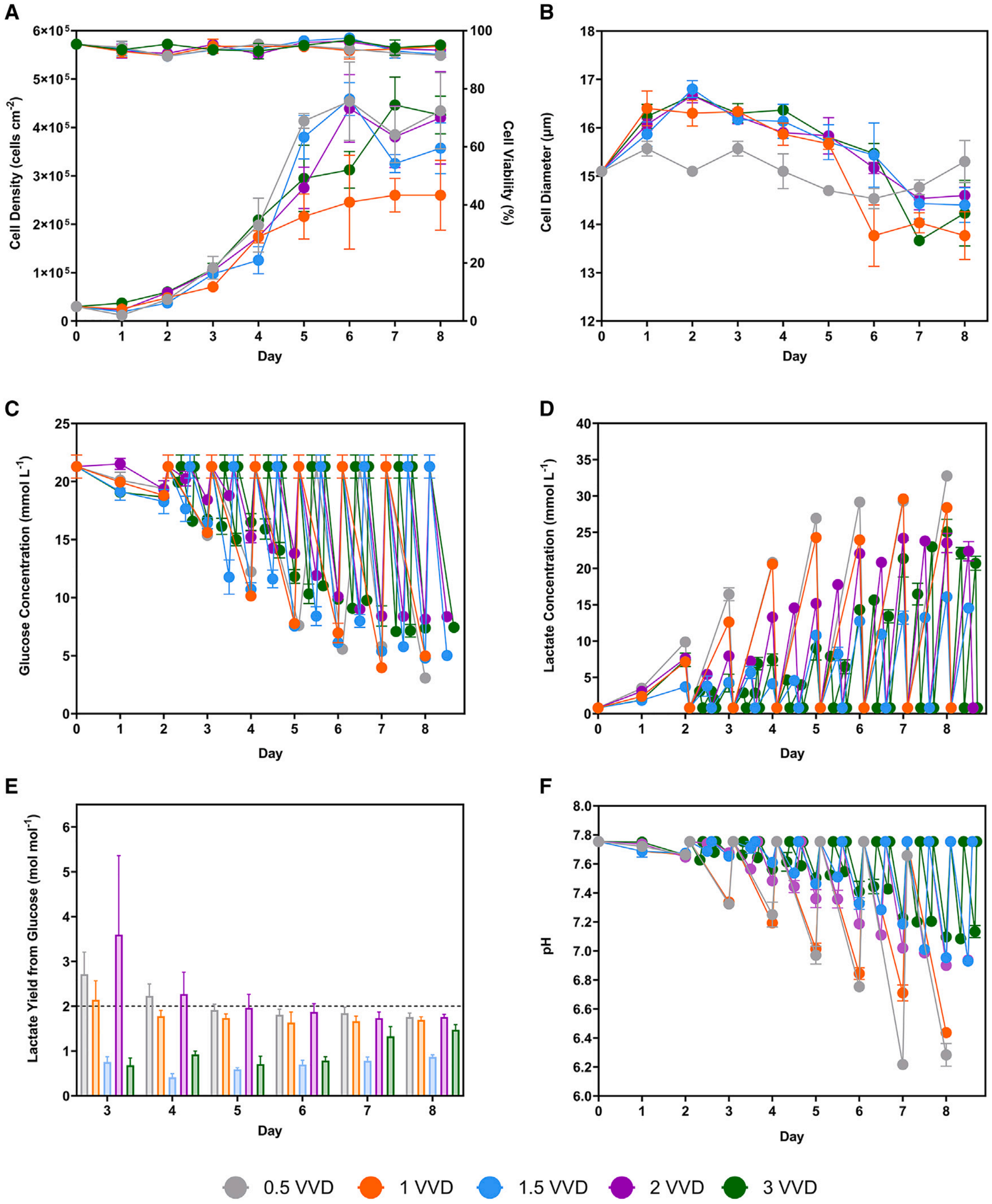

Figure 4. Cell culture profile during the quasi-perfusion study for LV production

(A) Viable WinPac-RDpro-GFP cell number and (B) cell diameter at each quasi-perfusion rate (n = 3). Daily offline measurement of (C) glucose and (D) lactate at each quasi-perfusion rate (n = 3). (F) The pH at quasi-perfusion rate (n = 1). (E) The lactate yield from glucose at each quasi-perfusion rate from days 3 to 8. Data points and bars represent mean value ± one standard deviation (N = 3). The 2 mol mol^{-1} reference line indicates the maximum theoretical yield of lactate from glucose during anaerobic glycolysis. WinPac-RDpro-GFP cells were seeded at 3×10^4 cells cm^{-2} and were expanded in T-25 flasks for 8 days with quasi-perfusion rates of 0.5, 1, 1.5, 2, and 3 VVD commencing 48 h after seeding.

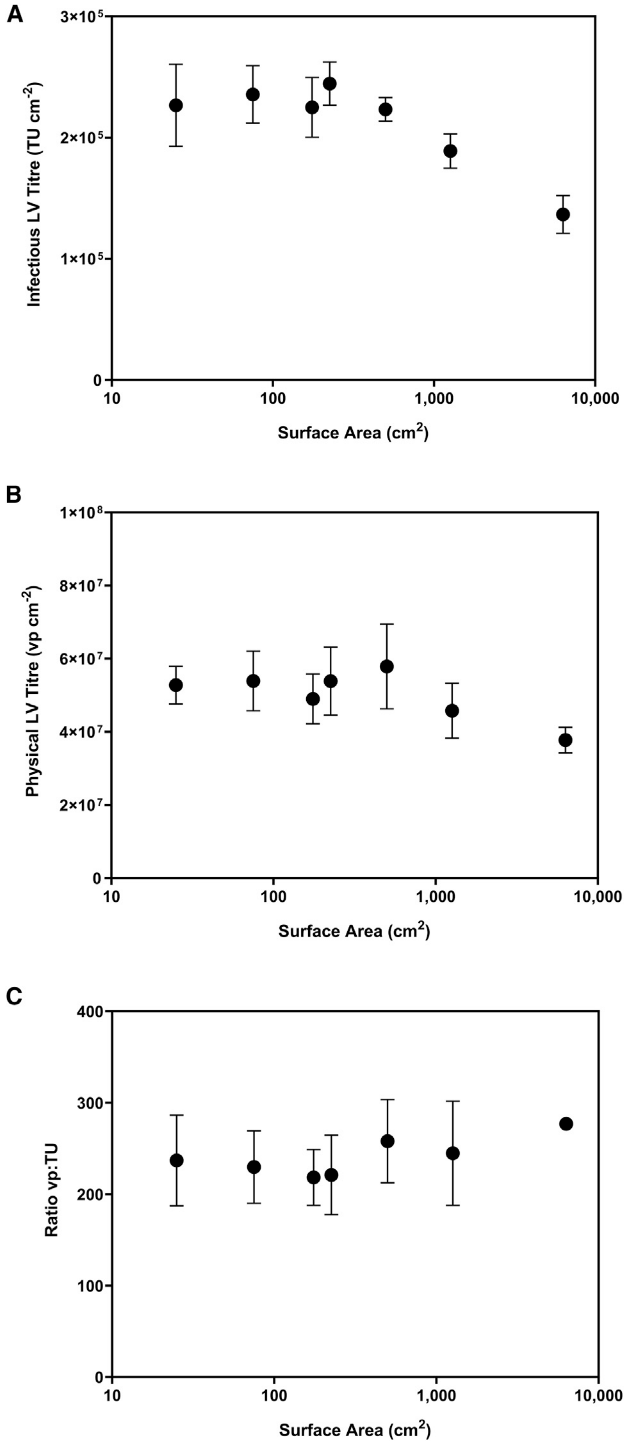

Figure 5. LV titres and product quality during scale-up experiment
(A) Infectious LV, (B) physical LV, and (C) ratio of physical-to-infectious LV produced in flasks with surface areas of 25, 75, 175, 225, 500, 1,264, and 6,320 cm^2 ($n = 3$). Flasks were seeded with 3×10^4 cells cm^{-2} WinPac-RDpro-GFP and expanded for 8 days, with quasi-perfusion at 1 VVD commencing 48 h after seeding. Points represent mean value ± one standard deviation ($N = 3$).

manufacture vectors with different transgenes and envelope proteins. However, this approach faces challenges such as batch-to-batch variability and the high cost of the plasmid DNA and transfection reagent.[6,8,29] There is interest in transitioning to stable producer cell lines to address these issues, facilitating reproducible, cost-effective, and scalable LV manufacturing.[8,9] This study established scalable manufacturing processes using a stable producer cell line and quasi-perfusion culture.

The WinPac-RDpro cell line constitutively expresses third-generation LVs with the RDpro envelope protein.[19] LV production processes using this cell line in standard and multilayer flasks reported seeding densities of 2×10^5 and 4.6×10^4–1.7×10^5 cells cm^{-2}, respectively.[19,29,30] The initial goal was to adjust the seeding density to match those reported for LV production processes in flasks and bioreactors.[20,21,23,24,31] Reducing the seeding density would facilitate scale-up by lowering the number of flasks requiring maintenance and processing time. Thus, this study evaluated the impact of seeding densities ranging from 1×10^4 and 5×10^4 cells cm^{-2} on cell growth kinetics, metabolite consumption and production, and LV titers.

The process was terminated on day 8 due to cell confluency and detachment from the culture vessel. The presence of producer cells in the culture supernatant is undesirable, as it can cause fouling and reduce throughputs during subsequent membrane filtration steps.[32,33] In the current study, we have shown the manufacture of LVs using the WinPac-RDpro-GFP cell line in the iCELLis Nano FBR for up to 8 days only so that comparisons can be made with the 8-day data from flasks. However, a recent study showed that the experiment in the iCELLis was extended over 10 days with no notable cell detachment.[10] This can be attributed to the cell being entrapped within the polyethylene terephthalate (PET) macrocarriers. The comparable specific growth rates observed across the seeding densities indicated that different seeding densities or conditions observed concerning metabolite concentrations or pH did not impact the cell growth. The specific growth rates were comparable with those observed when expanding the WinPac-RDpro-GFP cell line in the iCELLis Nano FBR, suggesting that the flasks were representative of cell growth on the PET macrocarriers and could be used in the initial process development (e.g., media screening) before transfer to the fixed bed bioreactor.[10]

Glucose undergoes various metabolic routes, with a significant proportion degraded via anaerobic glycolysis, resulting in lactate production.[34] Seeding densities between 2 and 5×10^4 cells cm^{-2} showed a continuous decrease in glucose concentrations until day 6, which followed the increase in cell density. The concentration stabilized beyond this point, which corresponded with confluency being achieved. Lactate concentrations exhibited a similar pattern, increasing until day 6, when they plateaued. In contrast, seeding at 1×10^4 cells cm^{-2} achieved a more gradual increase in lactate and a decrease in glucose, which aligned with the increase in viable cell density. The concentrations remained similar to fresh medium levels for the first 4 days, followed by an exponential increase/decrease. On day 8, the similar concentration across all seeding densities indicates

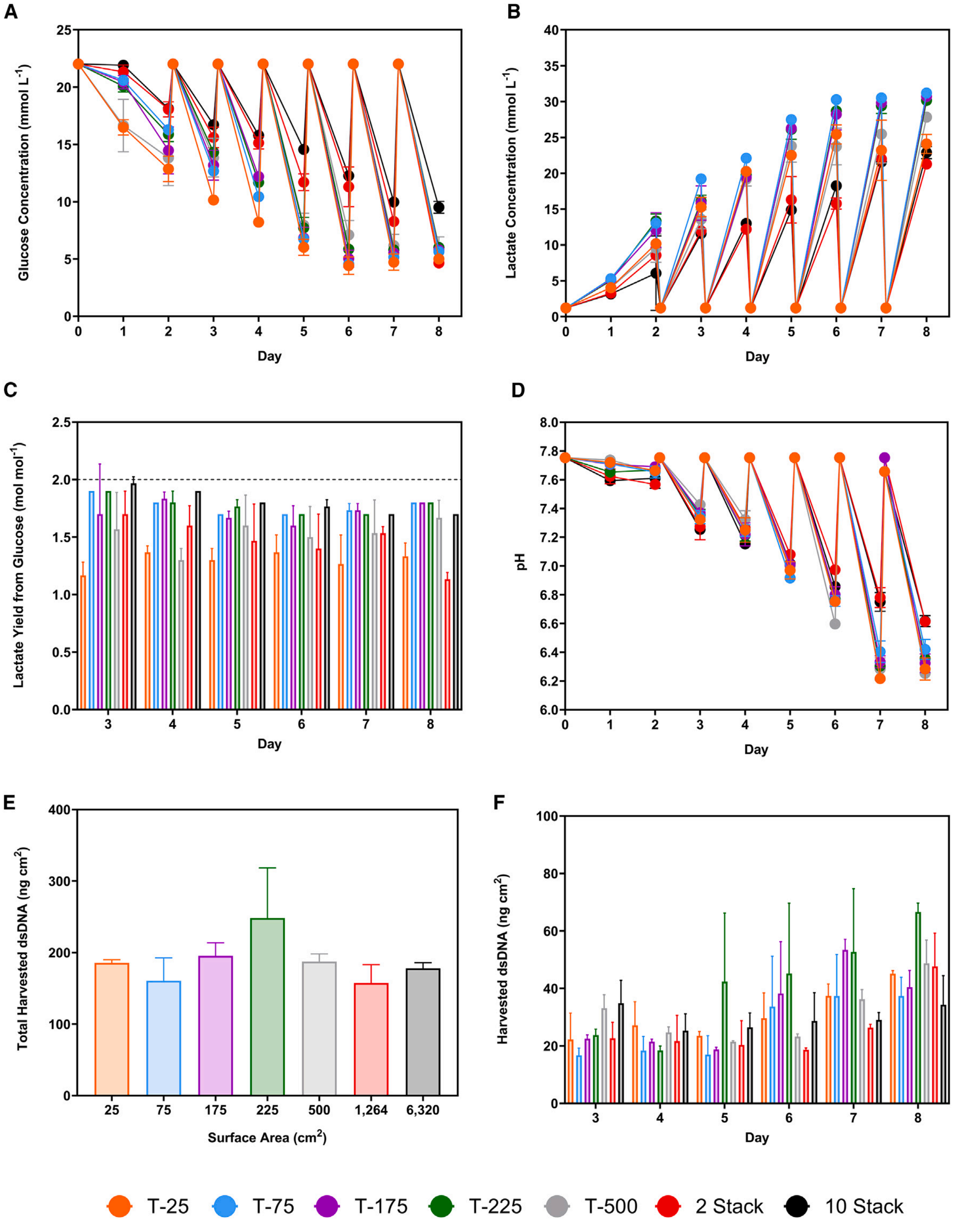

Figure 6. Cell culture profile during the scale-up experiment for LV production

Comparison of daily offline measurements of (A) glucose and (B) lactate ($n = 3$), and (D) offline pH ($n = 1$) for LV production in flasks with surface areas of 25, 75, 175, 225, 500, 1,264, and 6,320 cm^2. (C) The lactate yield from glucose at each seeding density from days 3 to 8. The 2 mol mol^{-1} reference line indicates the maximum theoretical yield of lactate from glucose during anaerobic glycolysis. (E) total dsDNA harvested and (F) dsDNA harvested daily across the LV production process in culture flasks with different surface areas ($n = 2$). WinPac-RDpro-GFP cells were seeded at 3×10^4 cells cm^{-2} and expanded for 8 days, with quasi-perfusion at 1 VVD commencing 48 h after seeding. Data points and bars represent mean value ± one standard deviation ($N = 3$).

Table 2. Comparison of cell growth kinetics and LV titers achieved in T-25 flasks (N = 3), multilayer flasks (N = 3) and the iCELLis Nano bioreactor (N = 1)

Culture vessel	Specific growth rate (h^{-1})	Doubling time (h)	Population doublings	Total TU cm^{-2}	Total vp cm^{-2}	vp:TU Ratio
T-25 Flask	0.024 ± 0.001	28.9 ± 1.3	3.3 ± 0.1	$(2.3 \pm 0.3) \times 10^5$	$(5.3 \pm 0.4) \times 10^7$	236 ± 40
Multilayer flask	NM	NM	NM	$(1.4 \pm 0.3) \times 10^5$	$(2.2 \pm 0.2) \times 10^7$	277 ± 6
iCELLis Nano Bioreactor (Quasi-Perfusion)[10]	0.016	44.2	3.3	3.1×10^4	7.3×10^6	238

WinPac-RDpro-GFP cells were seeded at 3×10^4 cells cm^{-2} and expanded for 8 days, with quasi-perfusion at a rate of 1 VVD commencing 48 h after seeding. The bioreactor run shown here are data from day 8 of the quasi-perfusion experiment at 1 VVD in our recent work.[10]
NM, not measured.

that the flasks supported the maximum number of metabolizing cells. Despite lactate levels exceeding 20 mmol L^{-1} from day 5 onward, they are not reportedly inhibitory to cell expansion.[35] The similar cell growth kinetics observed across the seeding densities demonstrate this. Glucose was not depleted during the process, indicating it was not limiting cell expansion.

The lactate yield from glucose provides insight into the efficiency of WinPac-RDpro-GFP cell metabolism. Oxidative phosphorylation is the most efficient mechanism of consuming glucose, producing 30–32 adenosine triphosphate (ATP) molecules per mole of glucose.[36] In contrast, anaerobic glycolysis yields only two ATP molecules and 2 moles of lactate per mole of glucose consumed.[37] The approximately 2 mol mol^{-1} yields indicated that the WinPac-RDpro-GFP cells were consuming glucose by anaerobic glycolysis. On day three, seeding densities of 2 and 3×10^4 cells cm^{-2} exceeded the maximum theoretical yield of lactate from glucose. This suggests that lactate production may have occurred through the catabolism of alternative carbon sources to glucose, such as glutamine or other amino acids.[37]

Ammonium accumulation can result from the chemical breakdown of glutamine or the enzymatic activity of fetal bovine serum (FBS).[38,39] Throughout the runs, the ammonium concentration peaked at seeding densities of 4 and 5×10^5 cells cm^{-2} at approximately 1.1 mmol L^{-1}, a previously non-inhibitory level for human embryonic kidney (HEK)293 cell growth.[34] This is supported by the similar cell growth kinetics observed across the seeding densities.

Lactate production during cell metabolism led to a decrease in pH across seeding densities. Previous studies have highlighted that mildly acidic culture conditions were associated with increased LV titers. Notably, improved infectious and physical LV titers have been reported for VSV-G, RDpro and gibbon-ape leukemia virus pseudotyped LVs produced at a pH of 6.0.[10,20,40]

Seeding densities of 3, 4, and 5×10^4 cells cm^{-2} showed similar total cell productivities of infectious LVs. The lower productivities observed at 1 and 2×10^4 cells cm^{-2} were possibly due to the higher culture pH observed. When producing LVs using the WinPac-RDpro-GFP cell line in the iCELLis Nano FBR, a sharp temporary increase in titers was seen when the pH was shifted from 7.20 to 6.85, indicating that this process parameter impacts the production.[10] The comparable total cell productivities of physical LVs indicated that the lower culture pH impacted infectious LV titers by either increasing production or stabilizing the vector. The 4×10^4 and 5×10^4 cells cm^{-2} seeding densities produced the best quality supernatant based on the ratio of physical-to-infectious LVs calculated using the specific productivities. Lower ratios of physical-to-infectious particles are more desirable due to a lower proportion of non-functional vectors.[41] The 3×10^4 cells cm^{-2} seeding density maximized infectious LV productivity and minimized the seeding density, so it was selected for further optimization. This experiment aimed to align the seeding density with reported values for LV production in FBRs. Reported seeding densities for viral vector production using HEK293T cells in FBRs range between 5×10^3 and 1.3×10^4 cells cm^{-2}.[20,21,23,24,31] Seeding at 3×10^4 cells cm^{-2} represents a significant reduction in comparison with the 2×10^5 and 4.6×10^4–1.7×10^5 cells cm^{-2} used in standard and multilayer flasks, respectively, to produce LVs with the WinPac-RDpro-GFP cells.[19,29,30] The lower seeding density facilitates scale-up by decreasing the number of flasks requiring maintenance during the seed train.

LVs are known to be unstable particles, with their half-lives influenced by temperature, exhibiting greater stability at lower temperatures.[42] Minimizing the vector's exposure to unfavorable temperatures was crucial to enhancing titers. Consequently, the vector's $t_{1/2}$ was determined at various temperatures. Consistent with previous research, a decrease in $t_{1/2}$ was observed as the incubation temperature increased.[42–44] The $t_{1/2}$ at 37°C aligned with a prior study, which reported 21.2 ± 8.6 h LVs pseudotyped with the RD114 envelope protein incubated under similar conditions, namely, DMEM supplemented with 10% FBS at 37°C[44]

Due to the low $t_{1/2}$ of LVs at 37°C, higher vector recoveries could be achieved by reducing the vector's exposure time to this temperature.[40,42] Increased quasi-perfusion rates could also enhance culture conditions by preventing nutrient depletion and minimizing the accumulation of inhibitory metabolites. Therefore, the impact of quasi-perfusion rate on cell growth kinetics, metabolite concentrations, and LV titers was investigated when seeding with 3×10^4 cells cm^{-2}.

The lowest specific growth rates were observed during the 0.5 and 1.5 VVD processes. In the case of the 0.5 VVD process, this could be attributed to either the depletion of essential nutrients necessary for cell proliferation or the accumulation of inhibitory metabolites. In

the 1.5 VVD process, where there was cell detachment from day 3, daily medium exchange involved a combination of partial and complete exchanges. However, no cell detachment was observed in the 3 VVD process, which included three complete medium exchanges per day, or in the 0.5 VVD process, with a single partial medium exchange. Based on this, it can be hypothesized that combining multiple medium exchanges, including a partial exchange, induced cell detachment. The specific growth rates for the 1, 2, and 3 VVD conditions were similar, suggesting that lactate concentrations greater than 20 mmol L^{-1} were not inhibitory to cell expansion. Similarly, the ammonium concentrations were also not found to be inhibitory.[34]

The glucose concentration profiles observed during the 1.5 VVD process showed similarities with those at 0.5 and 1 VVD, despite the notable difference in cell densities. It is postulated that the high glucose consumption levels could be due to a metabolic shift induced by cell detachment. As expected, the glucose concentrations remained higher in the 2 and 3 VVD processes due to the more frequent medium exchanges. Surprisingly, reducing the quasi-perfusion rate to 0.5 VVD did not produce higher lactate concentrations than the 1 VVD process. However, in contrast with the glucose consumption observation mentioned previously, the lactate concentrations during the 1.5 VVD process were lower than those observed for 0.5 and 1 VVD. This could indicate that the culture conditions induced a metabolic shift, lowering lactate production or consumption.

The cell metabolism was similar across the 0.5, 1, and 2 VVD processes, as indicated by the lactate yield from glucose being close to the theoretical yield of 2 mol mol^{-1} per day. However, in the 1.5 VVD process, lower lactate production was observed, resulting in a daily lactate yield from glucose of around 1 mol mol^{-1}. The 3 VVD process also exhibited a similar yield of approximately 1 mol mol^{-1}, which could be attributable to the culture conditions with a relatively high pH and levels of nutrients. Maintaining a similar lactate yield from glucose despite the evolving culture conditions, particularly the decrease in pH, sharply contrasted to that observed in the iCELLis Nano FBR with the same cell line.[10] After a decrease in pH from 7.20 to 6.85, the yield decreased to 0.45 ± 0.15 mol mol^{-1}. These differences in cell metabolism could be attributed to the pH decrease in flasks caused by lactate production, whereas the bioreactor adds carbon dioxide to decrease the pH.

The pH profiles of the 0.5 and 1 VVD processes showed comparable trends. This, coupled with the similar cell densities, indicates that the lower LV titers at 0.5 VVD can be attributed to the low vector stability. However, in the 1.5 VVD process, higher pH levels were observed, which can be attributed to the decreased viable cell density and, consequently, decreased lactate production. As mentioned above, this quasi-perfusion rate showed relatively low lactate concentration levels. Implementing increased quasi-perfusion rates prevented the culture pH from decreasing to less than 7.0 by minimizing lactate accumulation.

Decreasing the quasi-perfusion rate to 0.5 VVD resulted in a lower total infectious LV titer than the existing 1 VVD process. The extended time in the culture vessel at 0.5 VVD likely causes increased losses due to the low stability of the vector at 37°C. This aligns with the determined $t_{1/2}$ of the RDpro pseudotyped LVs, which was 16.6 ± 1.2 h at 37°C. Increasing the quasi-perfusion rates (1, 2, and 3 VVD) did not enhance the total cell productivities of infectious LV. The higher LV titers from the 1 VVD process can be attributed to the quasi-perfusion rate achieving reduced culture pH and harvesting the vector before losses through instability. Autotransduction—the phenomenon where the vector transduces the producer cell—could also cause losses in infectious LV. However, it is believed that autotransduction has minimal effect on RDpro-pseudotyped LVs as receptor occupancy by RDpro envelope proteins on the plasma membrane prevents attachment of vectors with the same receptor on producer cells, resulting in vector loss and/or cell entry.[45] The occurrence of autotransduction could be investigated using qPCR to calculate the copy number per cell for each integrated.[45] In contrast, at 2 and 3 VVD, the increased quasi-perfusion rates might have decreased the infectious LV yield due to elevated culture pH. LV entry into the host cells occurs after recognition between the vector and cell receptor, which causes a conformation change, which leads to direct fusion between the plasma and viral membranes. In the case of RDpro-pseudotyped LVs, this process is believed to be pH independent, and the fusion can occur at a neutral pH. Based on this, it can be postulated that the changing pH of the harvest medium did not impact infectivity.[46,47]

Across all five quasi-perfusion rates, the specific productivity of physical LV remained similar, supporting the hypothesis that culture pH influenced infectious LV yields. The 1 VVD process had the lowest physical-to-infectious LV particle ratio of 253 ± 5, consistent with bioreactor-based LV production processes using transfection and with continuous stable producer cell lines.[10,20,21]

The process with a seeding density of 3×10^4 cells cm^{-2} and a quasi-perfusion rate of 1 VVD was chosen for scale-up. In addition to maximizing the infectious LV titer and minimizing the ratio of physical-to-infectious LV, another consideration was maintaining low process volumes to facilitate DSP. The higher process volumes caused by increased VVDs will increase the hold and processing times of the initial DSP steps. Potential mitigation strategies involve continuously concentrating the harvested medium using single-pass tangential flow filtration.[48,49]

Manufacturing gene-modified cell therapies demands large quantities of high-quality LVs. To meet this demand, upstream production must be scaled up, or multiple sub-batches can be produced and pooled.[50] It is, therefore, critical that comparable LV titers, supernatant quality, and impurity levels be achieved when scaling up. This enables accurate prediction of the process scale required to achieve the desired LV quantities and facilitates DSP by providing consistent material for concentration and purification. When pooling, low batch-to-batch variability is critical to prevent the pooling of low-titer or quality sub-batches that could decrease the overall process yield.

Therefore, the implications of scaling up 3 $\times$ 10^4 cells cm^{-2} and 1 VVD process in flasks with increasingly large surface areas on LV titers and supernatant quality were investigated. Additionally, three independent processes were performed in each flask size to determine the impact of scale-up on batch-to-batch variability.

The differences observed in glucose concentrations between the multilayer flaks with surface areas of 1,264 cm^2 and 6,320 cm^2 and the smaller flasks indicated that the cells were not expanding at the same rate. A similar difference between the lactate concentrations was also observed. The analysis of lactate yield from glucose revealed a comparable daily value of 1.62 ± 0.24 mol mol^{-1}, indicating consistent cell metabolism across the vessels. As the cell metabolism was comparable across platforms, but the metabolite concentrations were different, it is postulated that there were fewer cells in the larger multilayer flask at a given time. This is likely due to suboptimal gas exchange between the layers of the larger multilayer flask compared with the smaller flask, which decreased the specific growth rate.

The process was successfully scaled from a 25 cm^2 flask to 1,264 cm^2, achieving comparable titers of physical and infectious LVs. Infectious LV titers were approximately 1.6-fold lower in the larger multilayer flask compared with the flasks with smaller surface areas. Similarly, the physical LV titers were lower. This lower LV production is attributed to the decreased cell density in the larger multilayer flask. Additionally, fewer cells metabolizing caused higher culture pH levels observed, impacting the titers, as discussed previously. In multilayer flasks, concerns about the physical and chemical environment heterogeneity and the uniformity of gas exchange across the layers could have caused the decreased cell growth rate.[51] Across all the flasks, a comparable ratio of physical-to-infectious LVs was achieved, indicating that high-quality vector supernatant could be produced after scaling up over 250-fold.

Three independent processes were performed across all the vessels to study the batch-to-batch variability. The infectious and physical titers CVs were 7.7% ± 2.6% and 11.9% ± 3.0%, comparable with those achieved for transient production of VSV-G pseudotyped LV in an FBR.[20] Additionally, the CVs observed across all flask sizes were comparable with those observed when producing LVs using the same cell line in an FBR of 6.4% and 10.0% for infectious and physical LV.[10] Unlike the flasks, the bioreactor processes maintained control of the dissolved oxygen (DO) concentration and culture pH. In contrast, high batch-to-batch variability was reported when producing alternatively pseudotyped LVs, including RDpro, using transient transfection in flasks.[29] Therefore, the low variability is attributed to using a stable producer cell line.[10] This is likely due to consistent cell growth in flasks, resulting in similar viable cell densities, metabolite concentrations, and culture pH. As the cells constitutively express LVs, provided the cell density and conditions are similar, comparable titers should be achieved.

Upstream processing can have a significant impact on the subsequent DSP steps. Along with maintaining vector titers, it is also critical that process scale-up does not increase the impurity burden on the DSP. As dsDNA is a major process-related impurity requiring removal during the DSP, its concentration was tracked during scale-up. In a manufacturing process using a stable cell line, the dsDNA's source is the culture medium's FBS supplementation.[52] An alternative source is intracellular dsDNA released after cell death, which the culture conditions could trigger. The comparable dsDNA concentrations achieved when scaling up will facilitate DSP process development by providing a predictable DNA load for the subsequent purification steps, such as anion exchange chromatography or tangential flow filtration. The harvest dsDNA concentrations were comparable with the continuous LV production process using the same cell line in the FBR.[10] This indicated the flow of the medium through the fixed-bed and stirrer bar did not impact the harvest dsDNA concentrations. Continuous stable producer cell lines have the advantage of not requiring the addition of an inducer, like tetracycline or doxycycline, for vector production, eliminating the need for subsequent removal during DSP.

FBRs offer an attractive platform for LV production, as many stable producer cell lines are adherent. Therefore, there is no need to adapt stable producer cells to grow in suspension from adherent culture, which is associated with significant losses in infectious titers.[53,54] The process was transferred from the culture flasks to the iCELLis Nano bioreactor by maintaining a constant seeding density of 3 $\times$ 10^4 cells cm^{-2} across the platforms and manually exchanging the medium at 1 VVD.

After process transfer, the ratio of physical-to-infectious LV particles was 238 in the FBR. This closely matched the value obtained in the flasks (241 ± 39), indicating that the bioreactor produced LV material of comparable quality. The infectious LV titers achieved in the unoptimized bioreactor runs were lower by approximately an order of magnitude compared with the T-25 flasks. This could also be attributed to the difference in conditions between the platforms, with the culture pH at 7.20 ± 0.05 and the DO set to 50% in the bioreactor, while the T-flasks pH was lower, reaching 6.22 ± 0.02. The studies on quasi-perfusion established here have paved the way for developing a perfusion process in the iCELLis Nano bioreactor, where it was shown that lowering the pH in the fixed-bioreactor in perfusion processes increases the infectious LV titer.[10] Transitioning to continuous operations in the bioreactor also showed further improvement in the quality of LVs as illustrated by the reduction in the ratio of physical-to-infectious particles from 238, in quasi-perfusion operations, to 181, in an 8-day, 1 VVD continuous process.[10] To further improve the process, strategies include reducing serum concentration in harvested medium to reduce costs and simplify DSP,[23] exploring harvesting in a medium with a lower serum concentration or serum-free medium,[21,22,41,55] and investigating the use of supplements like cholesterols and lipids to enhance LV production.[56,57]

This study developed a scalable LV production process using a continuous stable producer cell line. In the T-25 flasks, seeding density and quasi-perfusion rate were critical for maximizing infectious

LV titers. Seeding densities of less than 3×10^4 cells cm^{-2} resulted in lower titers, likely due to higher culture pH. At the same time, the 0.5 VVD process led to the lowest yields, possibly caused by vector losses at 37°C. Quasi-perfusion rates above 1 VVD did not increase yields, which can be attributed to the higher culture pHs. Scaling up from the T-25 flasks achieved comparable physical and infectious LV titers to flasks with surface areas of 1,264 cm^2. Above this surface area, infectious titer decreased by approximately 1.6-fold, possibly due to slower cell growth from suboptimal gas exchange in the layers. All platforms showed low batch-to-batch variability caused by consistent cell growth and similar cell densities, metabolite concentrations, and culture pHs in the vessels. The process was then transferred to a FBR to establish a quasi-perfusion process. The insights from this work on quasi-perfusion were used to establish a continuous LV process, which was subsequently improved by studying the impact of culture pH and perfusion rate on LV titers.[10]

MATERIALS AND METHODS

Cell culture

This work used WinPac-RDpro-GFP cells that constitutively express third-generation LVs with a GFP marker.[19] The WinPac-RDpro-GFP cells growth medium was DMEM modified with high glucose, GlutaMAX, and phenol red (Thermo Fisher Scientific) and supplemented with 10% volume/volume (v/v) FBS (Gibco, Thermo Fisher Scientific) in a humidified incubator at 37°C and 5% CO_2. Blasticidin, hygromycin, phleomycin, and puromycin (InvivoGen, Inc.) at working concentrations of 10, 100, 30, and 1 μg mL^{-1}, respectively, were added during cell expansion but were removed during LV production.

HEK293T cells (ATCC) were cultured at 37°C and 5% CO_2 in DMEM modified with high glucose, GlutaMAX, and phenol red supplemented with 10% (v/v) FBS.

Seeding density optimization

WinPac-RDpro-GFP cells were seeded in T-25 flasks (Thermo Fisher Scientific) at densities of 1, 2, 3, 4, and 5×10^4 cells cm^{-2} with 0.17 mL cm^{-2} of growth medium. Quasi-perfusion commenced 2 days after seeding for 6 days at 1 VVD. Each day, sacrificial sampling of three flasks was performed to determine the cell density, viability, and diameter. After harvesting, the infectious LV titer and offline pH were determined immediately, as described below. Samples were aliquoted from the remaining harvested medium and stored at −80°C for metabolite concentration analysis and physical LV titration.

Quasi-perfusion rate optimization

WinPac-RDpro-GFPcells were seeded at 3×10^4 cells cm^{-2} in T-25 flasks with 0.17 mL cm^{-2} of the growth medium. Quasi-perfusion commenced 2 days after seeding at rates of 0.5, 1, 1.5, 2, and 3 VVD for 6 days. Each day, sacrificial sampling of three flasks was performed to determine the cell density, viability, and diameter. After each medium exchange, the infectious titer and offline pH were determined immediately, as described below. Samples were aliquoted from the remaining harvested medium and stored at −80°C for metabolite concentration analysis and physical LV titration.

Scale-up of quasi-perfusion LV production process

WinPac-RDpro-GFP cells were seeded at 3×10^4 cells cm^{-2} in T-25, T-75, T-175, T-225 and T-500 flasks (Thermo Fisher Scientific) and Nunc Cell Factories (Thermo Fisher Scientific) with a surface area of 1,264 cm^2 and 6,320 cm^2 (Thermo Fisher Scientific) with 0.17 mL cm^{-2} of growth medium. Quasi-perfusion at a rate of 1 VVD commenced 2 days after seeding for 6 days. After the medium exchange, the offline pH was determined immediately each day, as described below. Samples were aliquoted from the remaining harvested medium and stored at −80°C for metabolite concentration analysis and infectious and physical LV titration.

LV production in iCELLis Nano bioreactor

Details of the experimental method using the FBR were described previously.[10] Only data up to day 8 at 1 VVD are shown in the current work to compare with the eight-day flask process. An iCELLis Nano bioreactor (Cytiva) was used with a 2-cm, low-compaction fixed bed (0.53 m^2 surface area) with the mPath bioreactor benchtop control tower (Cytiva). The vessel was filled with 600 mL growth medium and equilibrated overnight at 37°C with pH and DO setpoints of 7.20 ± 0.05 and 50% ± 2%, respectively. The next day, the bioreactor was inoculated at a seeding density of 3×10^4 cells cm^{-2}, and the total vessel volume was increased to 900 mL (0.17 mL cm^{-2}). The magnetic stirrer was set to achieve a linear speed of 2 cm s^{-1} to promote cell attachment. Once 80% of the seeded cells were attached, the linear speed was decreased to 1 cm s^{-1}. DO was monitored using a VisiFerm DO ECS 120 H0 (Hamilton Company, Inc.), and pH was maintained using carbon dioxide and 7.5% (v/v) sodium bicarbonate (Merck KGaA). Each day, 10 mL of the total bioreactor volume was removed, and pH measurements were performed to offset the online value, with the remaining medium aliquoted and stored at −80°C for metabolite concentration analysis. Three macrocarriers were sampled daily from the top of the fixed bed using autoclaved tweezers. Each microcarrier was placed in a 2-mL Eppendorf Tube (Eppendorf AG) with 1.5 mL lysis solution A100 (ChemoMetec A/S) and vortexed for 2 min for cell counting. Quasi-perfusion culture commenced two days post-seeding at a rate of 1 VVD. The harvested medium was collected, pooled, and stored at room temperature. Once per day, approximately 20 mL was removed from the pooled perfusate to determine the infectious titer and to aliquot and stored at −80°C to determine metabolite concentrations and physical titer.

Concurrent with the bioreactor runs, T-175 flasks (Thermo Fisher Scientific) were seeded with WinPac-RDpro-GFP cells at 3×10^4 cells cm^{-2} with 0.17 mL cm^{-2} of culture medium. Quasi-perfusion commenced 2 days after seeding at a rate of 1 VVD to simulate the medium exchange in the bioreactor. As with the bioreactor runs, the harvested medium was collected, pooled, and stored at room temperature, and the infectious titer was determined daily. The remaining medium was aliquoted and stored at −80°C for physical LV titer determination.

Determination of LV $t_{1/2}$

WinPac-RDpro-GFP cells were seeded in T-225 flasks (Thermo Fisher Scientific) at 3×10^4 cells cm^{-2} with 0.17 mL cm^{-2} of

the growth medium. A daily medium exchange commenced two days post-seeding. Vector-containing supernatant was harvested 6 days after seeding, pooled and clarified using a 0.45-μm Stericup (Merck KGaA). One milliliter of pooled supernatant was then aliquoted into 15-mL Falcon tubes (Thermo Fisher Scientific) and stored at either 4°C, 21°C, or 37°C. Every 24 h, three aliquots were taken at each temperature and assayed to determine the infectious titer as described below. The $t_{1/2}$ was determined using Equation 1:

$$N(t) = N_0\left(\frac{1}{2}\right)^{\frac{t}{t_{1/2}}}, \quad \text{(Equation 1)}$$

Where $t_{1/2}$ represents the vector half-life, N(t) and N_0 are the number of infectious LVs at time t and 0, respectively, and t represents the time elapsed in hours.

LV quantification

Infectious LV titers were determined using flow cytometric detection of GFP-positive cells. Quantification was performed with at least three technical repeats. Briefly, in 12-well plates (Thermo Fisher Scientific), HEK293T cells seeded at 3×10^5 cells $well^{-1}$ were transduced with neat LV samples in the presence of 8 μg mL^{-1} polybrene (Santa Cruz Biotechnology, Inc.) in a total of 500 μL. After 24 h of incubation at 37°C and 5% CO_2, 1 mL medium was added to each well. At 72 h after transduction, cells were trypsinized and stained with 7-AAD (Thermo Fisher Scientific) before flow cytometry using a BD LSRFortessa Cell Analyzer (Becton, Dickinson and Company). The gating approach used is shown in Figure S2. Infectious titers in transducing units per milliliter (TU mL^{-1}) were calculated from vector dilutions where 1–20% of the live cell population was GFP-positive using Equation 2:

$$Infectious\ titre\ (TU.mL^{-1}) = \left\{Number\ of\ cells\ at\ transduction \times \frac{(\%Live\ GFP - positive\ cells/100)}{Vector\ input\ volume}\right\} \times Dilution\ factor. \quad \text{(Equation 2)}$$

Physical vector particles per milliliter (vp mL^{-1}) were determined by measuring HIV-1 p24 capsid protein using an ELISA (OriGene Technologies, Inc.) using the recommended estimation of 10,000 physical LV particles per picogram of p24. Samples stored at −80°C were thawed and assayed with three technical repeats.

The CV was calculated using Equation 3, where σ represented the population's standard deviation, and μ is the population mean.

$$CV = \frac{\sigma}{\mu} \times 100 \quad \text{(Equation 3)}$$

Cell count, viability, and diameter measurement

Cell concentrations, viability, and diameter were determined using a NucleoCounter NC-200 system and Via1-Cassette (ChemoMetec A/S) using the viability and cell count assay protocol. Where necessary, cells were diluted using culture medium to obtain the recommended cell concentration between 5×10^4 and 5×10^6 cells mL^{-1}.

Specific growth rate (μ) was calculated using Equation 4, where Cx(t), and Cx(0) are the total cell numbers at the end and the start of the exponential growth phase, respectively. Time was t (h).

$$\mu = \frac{ln\left(\frac{Cx(t)}{Cx(0)}\right)}{\Delta t}. \quad \text{(Equation 4)}$$

The doubling time (t_d) was calculated using Equation 5, where μ represents the specific growth rate (h^{-1}).

$$t_d = \frac{ln(2)}{\mu}. \quad \text{(Equation 5)}$$

The number of population doublings was calculated using Equation 6, where Cx(t) and Cx(0) represented the total cell numbers at the end and the start of the exponential growth phase, respectively.

$$P_d = \frac{1}{ln(2)} \times ln\left(\frac{C_x(t)}{C_x(0)}\right). \quad \text{(Equation 6)}$$

Metabolite analysis

Samples were retained in triplicate and stored at −80°C. The CuBiAn Bioanalyzer (Optocell GmbH & Co. KG) determined the concentrations of ammonium, glucose, and lactate. The system was operated according to the manufacturer's instructions.

The lactate yield from glucose ($Y_{Lac|Glc}$) was calculated using Equation 7, where Δ[Lac] and Δ[Glc] represented the lactate and glucose concentration variation, respectively, over the same time.

$$\frac{Y_{Lac}}{Y_{Glc}} = \frac{\Delta[Lac]}{\Delta[Glc]} \quad \text{(Equation 7)}$$

Offline pH measurement

After sampling, offline pH measurements were performed immediately using a SevenCompact pH meter S220 (Mettler Toledo, LLC).

dsDNA concentration determination

dsDNA was quantified using the Quant-iT PicoGreen dsDNA assay kit (Thermo Fisher Scientific). The λ dsDNA standard underwent dilution in 1× TE buffer, covering a concentration range of

0–1,000 ng mL^{-1}. In 96-well plates, a mixture of 100 μL Quant-iT PicoGreen dsDNA reagent and 100 μL of either the sample or standard was prepared, followed by a 5-min incubation at room temperature. Subsequently, fluorescence was measured at 480/520 nm using a CLARIOstar plate reader (BMG LABTECH GmbH). The determination of DNA concentrations was based on the standard curve generated, and all measurements were performed in duplicate.

Graphing and statistical analysis

GraphPad Prism v9 (GraphPad Software, LLC) was used for graphical representation. In-text values are reported as the mean ± 1 SD. Statistical analysis was performed using IBM SPSS Statistics (IBM). Data was analyzed using a one-way ANOVA. Data that did not meet the requirements for a parametric test were analyzed using a Kruskal-Wallis one-way ANOVA. Statistical significance was defined as follows: $*p \leq 0.05$, $**p \leq 0.01$, $***p \leq 0.001$, $****p \leq$ or 0.0001.

DATA AND CODE AVAILABILITY

The data supporting this study's findings are available from the corresponding author upon reasonable request.

SUPPLEMENTAL INFORMATION

Supplemental information can be found online at https://doi.org/10.1016/j.omtm.2024.101264.

ACKNOWLEDGMENTS

The authors acknowledge the funding and support of the University College London – Cytiva Centre of Excellence, the UKRI Engineering and Physical Sciences Research Council (EPSRC) grant EP/L01520X/1, and the UKRI - Biotechnology and Biological Sciences Research Council BB/W014599/1. The authors thank Christopher Perry, Carme Ripoll Fiol, David Mainwaring, and Shahin Heshmatifar for their input.

AUTHOR CONTRIBUTIONS

Conceptualization: D.J.S., N.B.J., and A.C.M.E.R.; formal analysis: D.J.S.; funding acquisition: N.B.J. and A.C.M.E.R.; investigation: D.J.S. and P.S.C.; methodology: D.J.S, and P.S.C.; supervision: N.B.J., Q.A.R., and A.C.M.E.R.; visualization: D.J.S.; writing – original draft: D.J.S.; writing – review and editing: D.J.S., P.S.C., Y.T., Q.A.R., N.B.J., and A.C.M.E.R. All authors have read and agreed to the published version of the manuscript.

DECLARATION OF INTERESTS

D.J.S., P.S.C., Q.A.R., Y.T., and A.C.M.E.R. have no conflicts of interest. The work was funded, in part, by the UCL-Cytiva Centre of Excellence, as noted in the acknowledgements. N.B. Jackson was an employee of Cytiva at the time of submission.

REFERENCES

1. Milone, M.C., and O'Doherty, U. (2018). Clinical use of lentiviral vectors. Leukemia *32*, 1529–1541. https://doi.org/10.1038/s41375-018-0106-0.
2. Couto, P.S., Stibbs, D.J., Rotondi, M.C., Takeuchi, Y., and Rafiq, Q.A. (2023). Scalable manufacturing of gene-modified human mesenchymal stromal cells with microcarriers in spinner flasks. Appl. Microbiol. Biotechnol. *107*, 5669–5685. https://doi.org/10.1007/s00253-023-12634-w.
3. Aiuti, A., Biasco, L., Scaramuzza, S., Ferrua, F., Cicalese, M.P., Baricordi, C., Dionisio, F., Calabria, A., Giannelli, S., Castiello, M.C., et al. (2013). Lentiviral hematopoietic stem cell gene therapy in patients with Wiskott-Aldrich syndrome. Science *341*, 1233151. https://doi.org/10.1126/SCIENCE.1233151.
4. De Ravin, S.S., Wu, X., Moir, S., Anaya-O'Brien, S., Kwatemaa, N., Littel, P., Theobald, N., Choi, U., Su, L., Marquesen, M., et al. (2016). Lentiviral hematopoietic stem cell gene therapy for X-linked severe combined immunodeficiency. Sci. Transl. Med. *8*, 335ra57. https://doi.org/10.1126/scitranslmed.aad8856.
5. Cavazzana-Calvo, M., Payen, E., Negre, O., Wang, G., Hehir, K., Fusil, F., Down, J., Denaro, M., Brady, T., Westerman, K., et al. (2010). Transfusion independence and HMGA2 activation after gene therapy of human β-thalassaemia. Nature *467*, 318–322. https://doi.org/10.1038/NATURE09328.
6. Comisel, R.M., Kara, B., Fiesser, F.H., and Farid, S.S. (2021). Lentiviral vector bioprocess economics for cell and gene therapy commercialization. Biochem. Eng. J. *167*, 107868. https://doi.org/10.1016/j.bej.2020.107868.
7. Merten, O.W., Hebben, M., and Bovolenta, C. (2016). Production of lentiviral vectors. Mol. Ther. Methods Clin. Dev. *3*, 16017. https://doi.org/10.1038/mtm.2016.17.
8. Park, J., Inwood, S., Kruthiventi, S., Jenkins, J., Shiloach, J., and Betenbaugh, M. (2018). Progressing from transient to stable packaging cell lines for continuous production of lentiviral and gammaretroviral vectors. Curr. Opin. Chem. Eng. *22*, 128–137. https://doi.org/10.1016/J.COCHE.2018.09.007.
9. Ferreira, M.V., Cabral, E.T., and Coroadinha, A.S. (2021). Progress and perspectives in the development of lentiviral vector producer cells. Biotechnol. J. *16*, 2000017. https://doi.org/10.1002/BIOT.202000017.
10. Stibbs, D.J., Silva Couto, P., Takeuchi, Y., Rafiq, Q.A., Jackson, N.B., and Rayat, A.C.M.E. (2024). Continuous Manufacturing of Lentiviral Vectors using a Stable Producer Cell Line in a Fixed-Bed Bioreactor. Mol. Ther. Methods Clin. Dev. *32*, 101209. https://doi.org/10.1016/j.omtm.2024.101209.
11. Farson, D., Witt, R., McGuinness, R., Dull, T., Kelly, M., Song, J., Radeke, R., Bukovsky, A., Consiglio, A., and Naldini, L. (2001). A new-generation stable inducible packaging cell line for lentiviral vectors. Hum. Gene Ther. *12*, 981–997. https://doi.org/10.1089/104303401750195935.
12. Milani, M., Annoni, A., Bartolaccini, S., Biffi, M., Russo, F., Di Tomaso, T., Raimondi, A., Lengler, J., Holmes, M.C., Scheiflinger, F., et al. (2017). Genome editing for scalable production of alloantigen-free lentiviral vectors for *in vivo* gene therapy. EMBO Mol. Med. *9*, 1558–1573. https://doi.org/10.15252/emmm.201708148.
13. Klages, N., Zufferey, R., and Trono, D. (2000). A stable system for the high-titer production of multiply attenuated lentiviral vectors. Mol. Ther. *2*, 170–176. https://doi.org/10.1006/mthe.2000.0103.
14. Kafri, T., van Praag, H., Ouyang, L., Gage, F.H., and Verma, I.M. (1999). A packaging cell line for lentivirus vectors. J. Virol. *73*, 576–584.
15. Cockrell, A.S., Ma, H., Fu, K., McCown, T.J., and Kafri, T. (2006). A trans-lentiviral packaging cell line for high-titer conditional self-inactivating HIV-1 vectors. Mol. Ther. *14*, 276–284. https://doi.org/10.1016/j.ymthe.2005.12.015.
16. Xu, K., Ma, H., McCown, T.J., Verma, I.M., and Kafri, T. (2001). Generation of a stable cell line producing high-titer self-inactivating lentiviral vectors. Mol. Ther. *3*, 97–104. https://doi.org/10.1006/mthe.2000.0238.
17. Stewart, H.J., Leroux-Carlucci, M.A., Sion, C.J.M., Mitrophanous, K.A., and Radcliffe, P.A. (2009). Development of inducible EIAV-based lentiviral vector packaging and producer cell lines. Gene Ther. *16*, 805–814. https://doi.org/10.1038/gt.2009.20.
18. Stewart, H.J., Fong-Wong, L., Strickland, I., Chipchase, D., Kelleher, M., Stevenson, L., Thoree, V., McCarthy, J., Ralph, G.S., Mitrophanous, K.A., and Radcliffe, P.A. (2011). A stable producer cell line for the manufacture of a lentiviral vector for gene therapy of parkinson's disease. Hum. Gene Ther. *22*, 357–369. https://doi.org/10.1089/hum.2010.142.
19. Sanber, K.S., Knight, S.B., Stephen, S.L., Bailey, R., Escors, D., Minshull, J., Santilli, G., Thrasher, A.J., Collins, M.K., and Takeuchi, Y. (2015). Construction of stable packaging cell lines for clinical lentiviral vector production. Sci. Rep. *5*, 9021. https://doi.org/10.1038/srep09021.

20. Valkama, A.J., Leinonen, H.M., Lipponen, E.M., Turkki, V., Malinen, J., Heikura, T., Ylä-Herttuala, S., and Lesch, H.P. (2018). Optimization of lentiviral vector production for scale-up in fixed-bed bioreactor. Gene Ther. *25*, 39–46. https://doi.org/10.1038/gt.2017.91.

21. Leinonen, H.M., Lepola, S., Lipponen, E.M., Heikura, T., Koponen, T., Parker, N., Ylä-Herttuala, S., and Lesch, H.P. (2020). Benchmarking of Scale-X bioreactor system in lentiviral and adenoviral vector production. Hum. Gene Ther. *31*, 376–384. https://doi.org/10.1089/hum.2019.247.

22. Leinonen, H.M., Lipponen, E.M., Valkama, A.J., Hynynen, H., Oruetxebarria, I., Turkki, V., Olsson, V., Kurkipuro, J., Samaranayake, H., Määttä, A.-M., et al. (2019). Preclinical Proof-of-Concept, Analytical Development, and Commercial Scale Production of Lentiviral Vector in Adherent Cells. Mol. Ther. Methods Clin. Dev. *15*, 63–71. https://doi.org/10.1016/j.omtm.2019.08.006.

23. Powers, A.D., Drury, J.E., Hoehamer, C.F., Lockey, T.D., and Meagher, M.M. (2020). Lentiviral vector production from a stable packaging cell line using a packed bed bioreactor. Mol. Ther. Methods Clin. Dev. *19*, 1–13. https://doi.org/10.1016/j.omtm.2020.08.010.

24. Fiol, C.R., Collignon, M.L., Welsh, J., and Rafiq, Q.A. (2023). Optimizing and developing a scalable, chemically defined, animal component-free lentiviral vector production process in a fixed-bed bioreactor. Mol. Ther. Methods Clin. Dev. *30*, 221–234. https://doi.org/10.1016/j.omtm.2023.06.011.

25. Sena-Esteves, M., Tebbets, J.C., Steffens, S., Crombleholme, T., and Flake, A.W. (2004). Optimized large-scale production of high titer lentivirus vector pseudotypes. J. Virol. Methods *122*, 131–139. https://doi.org/10.1016/j.jviromet.2004.08.017.

26. Rout-Pitt, N., McCarron, A., McIntyre, C., Parsons, D., and Donnelley, M. (2018). Large-scale production of lentiviral vectors using multilayer cell factories. J. Biol. Methods *5*, 90. https://doi.org/10.14440/jbm.2018.236.

27. Merten, O.W., Charrier, S., Laroudie, N., Fauchille, S., Dugué, C., Jenny, C., Audit, M., Zanta-Boussif, M.A., Chautard, H., Radrizzani, M., et al. (2011). Large-scale manufacture and characterization of a lentiviral vector produced for clinical ex vivo gene therapy application. Hum. Gene Ther. *22*, 343–356. https://doi.org/10.1089/hum.2010.060.

28. Lesch, H.P., Valonen, P., and Karhinen, M. (2021). Evaluation of the Single-Use Fixed-Bed Bioreactors in Scalable Virus Production. Biotechnol. J. *16*, e2000020. https://doi.org/10.1002/BIOT.202000020.

29. Perry, C., Mujahid, N., Takeuchi, Y., and Rayat, A.C.M.E. (2023). Insights into product and process related challenges of lentiviral vector bioprocessing. Biotechnol. Bioeng. 1–16. https://doi.org/10.1002/bit.28498.

30. Ruscic, J., Perry, C., Mukhopadhyay, T., Takeuchi, Y., and Bracewell, D.G. (2019). Lentiviral vector purification using nanofiber ion-exchange chromatography. Mol. Ther. Methods Clin. Dev. *15*, 52–62. https://doi.org/10.1016/j.omtm.2019.08.007.

31. Lesch, H.P., Heikkilä, K.M., Lipponen, E.M., Valonen, P., Müller, A., Räsänen, E., Tuunanen, T., Hassinen, M.M., Parker, N., Karhinen, M., et al. (2015). Process Development of Adenoviral Vector Production in Fixed Bed Bioreactor: From Bench to Commercial Scale. Hum. Gene Ther. *26*, 560–571. https://doi.org/10.1089/hum.2015.081.

32. Greene, M.R., Lockey, T., Mehta, P.K., Kim, Y.S., Eldridge, P.W., Gray, J.T., and Sorrentino, B.P. (2012). Transduction of human CD34+ repopulating cells with a self-inactivating lentiviral vector for SCID-X1 produced at clinical scale by a stable cell line. Hum. Gene Ther. Methods *23*, 297–308. https://doi.org/10.1089/hgtb.2012.150.

33. Valkama, A.J., Oruetxebarria, I., Lipponen, E.M., Leinonen, H.M., Käyhty, P., Hynynen, H., Turkki, V., Malinen, J., Miinalainen, T., Heikura, T., et al. (2020). Development of Large-Scale Downstream Processing for Lentiviral Vectors. Mol. Ther. Methods Clin. Dev. *17*, 717–730. https://doi.org/10.1016/j.omtm.2020.03.025.

34. Hassell, T., Gleave, S., and Butler, M. (1991). Growth inhibition in animal cell culture. The effect of lactate and ammonia. Appl. Biochem. Biotechnol. *30*, 29–41. https://doi.org/10.1007/BF02922022.

35. Garnier, A., Côté, J., Nadeau, I., Kamen, A., and Massie, B. (1994). Scale-up of the adenovirus expression system for the production of recombinant protein in human 293S cells. Cytotechnology *15*, 145–155. https://doi.org/10.1007/BF00762389.

36. Wilson, D.F. (2017). Oxidative phosphorylation: regulation and role in cellular and tissue metabolism. J. Physiol. *595*, 7023–7038. https://doi.org/10.1113/JP273839.

37. Glacken, M.W. (1988). Catabolic control of mammalian cell culture. Nat. Biotechnol. *69*, 1041–1050. https://doi.org/10.1038/nbt0988-1041.

38. Tritsch, G.L., and Moore, G.E. (1962). Spontaneous decomposition of glutamine in cell culture media. Exp. Cell Res. *28*, 360–364. https://doi.org/10.1016/0014-4827(62)90290-2.

39. Wein, J., and Goetz, I.E. (1973). Asparaginase and glutaminase activities in culture media containing dialyzed fetal calf serum. In Vitro *9*, 186–193. https://doi.org/10.1007/BF02618436.

40. Holic, N., Seye, A.K., Majdoul, S., Martin, S., Merten, O.W., Galy, A., and Fenard, D. (2014). Influence of mildly acidic pH conditions on the production of lentiviral and retroviral vectors. Hum. Gene Ther. Clin. Dev. *25*, 178–185. https://doi.org/10.1089/HUMC.2014.027.

41. McCarron, A., Donnelley, M., McIntyre, C., and Parsons, D. (2019). Transient lentiviral vector production using a packed-bed bioreactor system. Hum. Gene Ther. Methods *30*, 93–101. https://doi.org/10.1089/hgtb.2019.038.

42. Higashikawa, F., and Chang, L. (2001). Kinetic analyses of stability of simple and complex retroviral vectors. Virology *280*, 124–131. https://doi.org/10.1006/viro.2000.0743.

43. Carmo, M., Alves, A., Rodrigues, A.F., Coroadinha, A.S., Carrondo, M.J.T., Alves, P.M., and Cruz, P.E. (2009). Stabilization of gammaretroviral and lentiviral vectors: from production to gene transfer. J. Gene Med. *11*, 670–678. https://doi.org/10.1002/jgm.1353.

44. Dautzenberg, I.J.C., Rabelink, M.J.W.E., and Hoeben, R.C. (2021). The stability of envelope-pseudotyped lentiviral vectors. Gene Ther. *28*, 89–104. https://doi.org/10.1038/s41434-020-00193-y.

45. Tijani, M., Munis, A.M., Perry, C., Sanber, K., Ferraresso, M., Mukhopadhyay, T., Themis, M., Nisoli, I., Mattiuzzo, G., Collins, M.K., and Takeuchi, Y. (2018). Lentivector Producer Cell Lines with Stably Expressed Vesiculovirus Envelopes. Mol. Ther. Methods Clin. Dev. *10*, 303–312. https://doi.org/10.1016/J.OMTM.2018.07.013.

46. Cosset, F.-L., Marianneau, P., Verney, G., Gallais, F., Tordo, N., Pécheur, E.-I., ter Meulen, J., Deubel, V., and Bartosch, B. (2009). Characterization of Lassa Virus Cell Entry and Neutralization with Lassa Virus Pseudoparticles. J. Virol. *83*, 3228–3237. https://doi.org/10.1128/jvi.01711-08.

47. McClure, M.O., Sommerfelt, M.A., Marsh, M., and Weiss, R.A. (1990). The pH independence of mammalian retrovirus infection. J. Gen. Virol. *71*, 767–773. https://doi.org/10.1099/0022-1317-71-4-767.

48. Chaubal, A.S., and Zydney, A.L. (2023). Single-pass tangential flow filtration (SPTFF) of nanoparticles: Achieving sustainable operation with dilute colloidal suspensions for gene therapy applications. Membranes *13*, 433. https://doi.org/10.3390/MEMBRANES13040433/S1.

49. Casey, C., Gallos, T., Alekseev, Y., Ayturk, E., and Pearl, S. (2011). Protein concentration with single-pass tangential flow filtration (SPTFF). J. Membr. Sci. *384*, 82–88. https://doi.org/10.1016/J.MEMSCI.2011.09.004.

50. Ausubel, L.J., Hall, C., Sharma, A., Shakeley, R., Lopez, P., Quezada, V., Couture, S., Laderman, K., McMahon, R., Huang, P., et al. (2012). Production of CGMP-Grade Lentiviral Vectors. Bioprocess Int. *10*, 32–43.

51. Rafiq, Q.A., Coopman, K., and Hewitt, C.J. (2013). Scale-up of human mesenchymal stem cell culture: current technologies and future challenges. Curr. Opin. Chem. Eng. *2*, 8–16. https://doi.org/10.1016/j.coche.2013.01.005.

52. Segura, M.d.l.M., Kamen, A., Trudel, P., and Garnier, A. (2005). A novel purification strategy for retrovirus gene therapy vectors using heparin affinity chromatography. Biotechnol. Bioeng. *90*, 391–404. https://doi.org/10.1002/bit.20301.

53. Guy, H.M., McCloskey, L., Lye, G.J., Mitrophanous, K.A., and Mukhopadhyay, T.K. (2013). Characterization of Lentiviral Vector Production Using Microwell Suspension Cultures of HEK293T-Derived Producer Cells. Hum. Gene Ther. Methods *24*, 125–139. https://doi.org/10.1089/hgtb.2012.200.

54. Segura, M.M., Garnier, A., Durocher, Y., Coelho, H., and Kamen, A. (2007). Production of lentiviral vectors by large-scale transient transfection of suspension cultures and affinity chromatography purification. Biotechnol. Bioeng. *98*, 789–799. https://doi.org/10.1002/bit.21467.

55. Sheu, J., Beltzer, J., Fury, B., Wilczek, K., Tobin, S., Falconer, D., Nolta, J., and Bauer, G. (2015). Large-scale production of lentiviral vector in a closed system hollow fiber bioreactor. Mol. Ther. Methods Clin. Dev. *2*, 15020. https://doi.org/10.1038/mtm.2015.20.

56. Mitta, B., Rimann, M., and Fussenegger, M. (2005). Detailed design and comparative analysis of protocols for optimized production of high-performance HIV-1-derived lentiviral particles. Metab. Eng. *7*, 426–436. https://doi.org/10.1016/J.YMBEN.2005.06.006.

57. Chen, M.-Y., Lie, P.-C., Li, Z.-L., and Wei, X. (2009). Endothelial differentiation of wharton s jelly derived mesenchymal stem cells in comparison with bone marrow derived mesenchymal stem cells. Exp. Hematol. *37*, 629–640. https://doi.org/10.1016/j.exphem.2009.02.003.

OMTM, Volume *32*

Supplemental information

Quasi-perfusion studies for intensified lentiviral vector production using a continuous stable producer cell line

Dale J. Stibbs, Pedro Silva Couto, Yasuhiro Takeuchi, Qasim A. Rafiq, Nigel B. Jackson, and Andrea C. M. E. Rayat

(A)

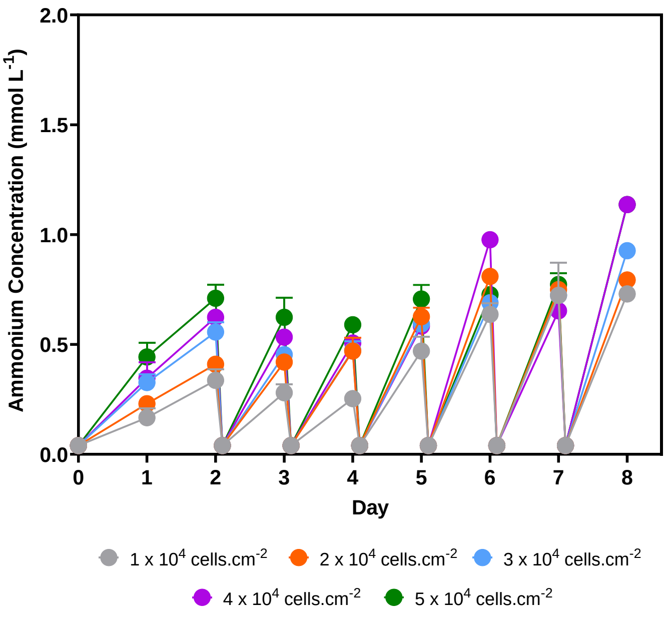

Ammonium Concentration (mmol L^{-1})
2.0
1.5
1.0
0.5
0.0
0
1
2
3
4
5
6
7
8
Day
1 x 10^4 cells.cm^{-2}
2 x 10^4 cells.cm^{-2}
3 x 10^4 cells.cm^{-2}
4 x 10^4 cells.cm^{-2}
5 x 10^4 cells.cm^{-2}

(B)

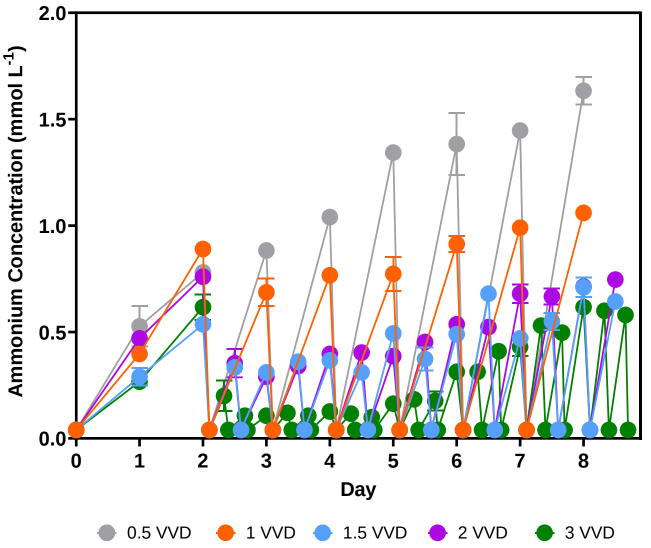

Ammonium Concentration (mmol L^{-1})
2.0
1.5
1.0
0.5
0.0
0
1
2
3
4
5
6
7
8
Day
0.5 VVD
1 VVD
1.5 VVD
2 VVD
3 VVD

(C)

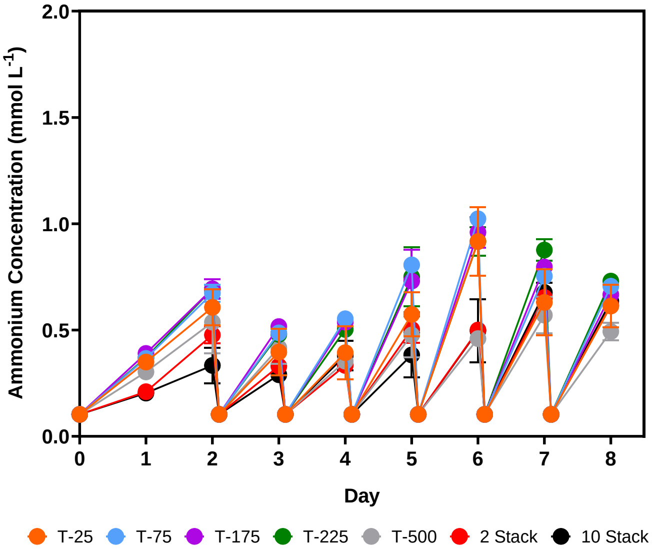

Ammonium Concentration (mmol L^{-1})
2.0
1.5
1.0
0.5
0.0
0
1
2
3
4
5
6
7
8
Day
T-25
T-75
T-175
T-225
T-500
2 Stack
10 Stack

Figure S1 – Comparison of daily off-line measurements of ammonia during **(A)** the seeding density experiment where WinPac-RDpro-GFP cells were seeded at 1, 2, 3, 4 and 5 x 10^4 cells cm^{-2} and expanded in T-25 flasks for 8 days with a quasi-perfusion rate of 1 VVD commencing 48 hours post-seeding, **(B)** the quasi-perfusion experiment where WinPac-RDpro-GFP cells were seeded at 3 x 10^4 cells cm^{-2} were expanded in T-25 flasks for eight days with quasi-perfusion rates of 0.5, 1, 1.5, 2 and 3 VVD commencing 48 hours post-seeding and **(C)** the scaling experiment where WinPac-RDpro-GFP cells were seeded at 3 x 10^4 cells cm^{-2} were expanded in T-25, T-75, T-175, T-225, T-500 flasks and 2- and 10-layer multilayer flasks for eight days with quasi-perfusion rates of 0.5, 1, 1.5, 2 and 3 VVD commencing 48 hours post-seeding. Points represent mean value ± one standard deviation (N = 3).

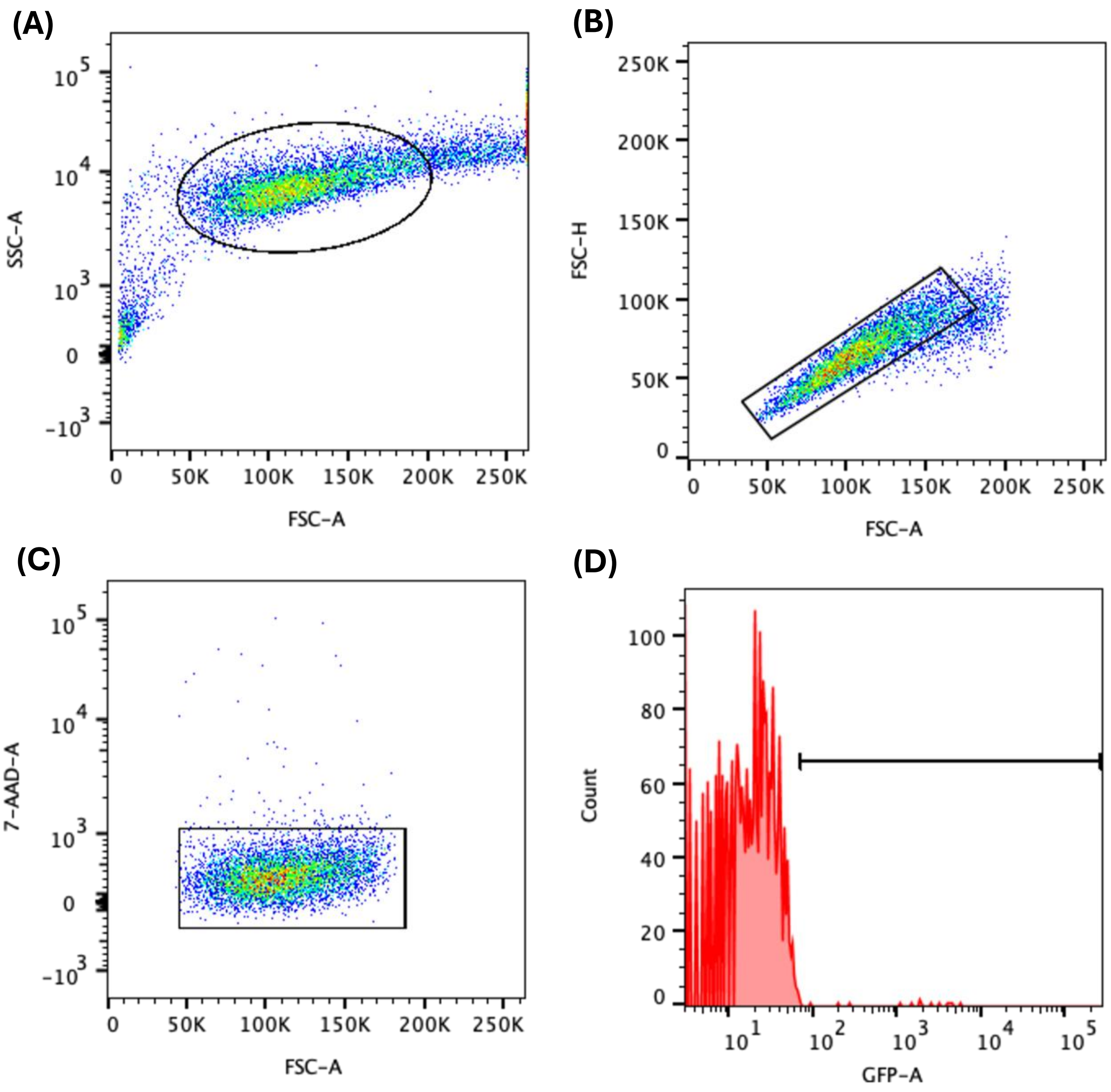

Figure S2 – Gating strategy for determining the percentage of GFP-expressing cells when tittering LVs using HEK 293T cells. **(A)** Gating of cells from debris; **(B)** Gating of singlets and doublets; **(C)** Gating of live cells through staining with 7-AAD; and **(D)** Gating of GFP-expressing cells.
